# A Survey of Computer-Aided Tumor Diagnosis Based on Convolutional Neural Network

**DOI:** 10.3390/biology10111084

**Published:** 2021-10-22

**Authors:** Yan Yan, Xu-Jing Yao, Shui-Hua Wang, Yu-Dong Zhang

**Affiliations:** School of Computing and Mathematical Sciences, University of Leicester, University Road, Leicester LE1 7RH, UK; yy284@leicester.ac.uk (Y.Y.); xy147@leicester.ac.uk (X.-J.Y.)

**Keywords:** tumor detection, convolutional neural network, application of tumor detection, traditional tumor detection methods, computer-aided diagnosis

## Abstract

**Simple Summary:**

One of the hottest areas in deep learning is computerized tumor diagnosis and treatment. The identification of tumor markers, the outline of tumor growth activity, and the staging of various tumor kinds are frequently included. There are several deep learning models based on convolutional neural networks that have high performance and accurate identification, with the potential to improve medical tasks. Breakthroughs and updates in computer algorithms and hardware devices, and intelligent algorithms applied in medical images have a diagnostic accuracy that doctors cannot match in some diseases. This paper reviews the progress of tumor detection from traditional computer-aided methods to convolutional neural networks and demonstrates the potential of the practical application of convolutional neural networks from practical cases to transform the detection model from experiment to clinical application.

**Abstract:**

Tumors are new tissues that are harmful to human health. The malignant tumor is one of the main diseases that seriously affect human health and threaten human life. For cancer treatment, early detection of pathological features is essential to reduce cancer mortality effectively. Traditional diagnostic methods include routine laboratory tests of the patient’s secretions, and serum, immune and genetic tests. At present, the commonly used clinical imaging examinations include X-ray, CT, MRI, SPECT scan, etc. With the emergence of new problems of radiation noise reduction, medical image noise reduction technology is more and more investigated by researchers. At the same time, doctors often need to rely on clinical experience and academic background knowledge in the follow-up diagnosis of lesions. However, it is challenging to promote clinical diagnosis technology. Therefore, due to the medical needs, research on medical imaging technology and computer-aided diagnosis appears. The advantages of a convolutional neural network in tumor diagnosis are increasingly obvious. The research on computer-aided diagnosis based on medical images of tumors has become a sharper focus in the industry. Neural networks have been commonly used to research intelligent methods to assist medical image diagnosis and have made significant progress. This paper introduces the traditional methods of computer-aided diagnosis of tumors. It introduces the segmentation and classification of tumor images as well as the diagnosis methods based on CNN to help doctors determine tumors. It provides a reference for developing a CNN computer-aided system based on tumor detection research in the future.

## 1. Introduction

### 1.1. The Health Hazards of Tumor

People’s living environment and dietary problems, such as chemical pollution in the industrial environment and reduced immunity caused by poor diet, may lead to various tumor diseases. There are many kinds of tumor diseases, including lung tumors, liver tumors, breast tumors, and many brain tumors, which seriously affect human health and threaten human life.

Tumor [1] refers to the new organisms that form a space-occupying lumpy protuberance formed by local tissue cell proliferation under the action of various tumorigenic factors [2]. According to the pathological morphology [3], growth mode, cellular characteristics of new organisms, and the degree of harm to the body, a tumor can be classified into malignant or benign. Malignant tumors can be classified as cancer and sarcoma. Malignant tumors originating from epithelial tissue are called “cancer” and those originating from mesenchymal tissue are called “sarcoma.” A malignant tumor—cancer—together with cardiovascular and cerebrovascular diseases and accidents, constitutes the top three causes of death in all countries worldwide. Figures provided by the World Health Organization show more than 3.7 million new cases of disease and about 1.9 million deaths each year [4]. An estimated 8.2 million people worldwide died from cancer in 2012, with 40 percent of cancer deaths attributed to smoking and excessive alcohol consumption [5]. Despite having some means of prevention, in Europe, which has only an eighth of the world’s population, there are about 3.7 million new cancer cases each year, accounting for 20 percent of deaths. Reducing exposure to common risk factors such as tobacco smoke can effectively prevent many cancers. In addition, most cancers can be cured with surgery, radiation, or chemotherapy. Therefore, early detection of pathological features is particularly significant to lessen cancer mortality rate effectively for the treatment of cancer.

In general, benign tumors in the human body are mainly due to local pressure, congestion, and organ function. Benign tumors grow slowly and generally do not affect the patient’s life. However, as the tumor grows, the compression of surrounding tissue is symptomatic. In particular, the compression of adjacent tissues causes a series of symptoms. Compression of the chest will lead to breathing difficulties, chest pain, cough, chest tightness, stomach compression, loss of appetite, nausea, and abdominal pain, distension, and vomiting. These symptoms can lead to clinical effects after certain radical surgery but malignant lesions may also occur, resulting in endocrine disorders, bleeding, and infection. Different from the former, malignant tumors have a severe impact on the body. The immature differentiation and rapid growth of malignant tumors will destroy the structure and function of organs, affecting the regular operation of the patient’s organs. If cough, hemoptysis, or even heart function is affected, the patient’s quality of life will also be seriously affected [6]. Progressive lesions of cancer at an advanced stage may lead to emaciation, weakness, anemia, or systemic failure. Malignant tumors increase and tend to metastasize, leading to other systemic syndromes and infections.

### 1.2. Methods of Tumor Diagnosis

Early tumors have no specific symptoms. Some symptoms sometimes accompany different types of tumors. It is possible to detect the growth of a malignant tumor early if the symptoms are detected in advance. When the possibility of a tumor is suspected, a comprehensive examination can be carried out to achieve a comprehensive and objective analysis of the tumor condition, carry out early treatment, and improve the cure rate.

Traditional diagnostic methods include routine laboratory examinations of the patient’s secretions and serum, immune, and genetic tests. Current commonly used clinical imaging examination includes X-ray, computerized tomography (CT), magnetic resonance imaging (MRI), ultrasound, single-photon emission computed tomography (SPECT) scan, etc. [7], and each method has its advantages [8]. CT can be used for space-occupying lesions of the head. However, in examining the skull and other brain tissues close to the bone wall, the CT imaging effect is not as good as MRI because of the interference of bone. SPECT examination can visually show the shape of the organs to determine whether there is a tumor in the organs. It is currently commonly used to evaluate the efficacy and diagnosis of bone tumors and bone metastases. MRI can diagnose a brain tumor, bone tumor, and so on, especially brain tumor diagnosis, and is obviously better than CT. However, the price of MRI examinations is relatively high, and the patients have a poor feeling in MRI examinations. Therefore, cancer can be generally judged by CT, and MRI examination is not necessary. Compared with X-ray, CT, MRI, and other examination methods, ultrasound examination is convenient and affordable. It does not rely on radiation, so patients do not have to worry about radiation damage [9].

Nevertheless, these medical imaging devices also have drawbacks. Due to the defects of medical imaging equipment and the complexity of human tissue, the original image information of human tissue collected by medical imaging equipment is limited. These images need some processing before they can be used as reference images to assist doctors in medical diagnosis. The use of medical imaging equipment in the process of medical images will produce certain radiation [10]. The radiation from medical scanning equipment can cause a certain amount of harm to doctors and patients and cause diseases like cancer. As a result, researchers have become interested in controlling and reducing radiation in the scans. However, with the emergence of new problems of radiation reduction, the excessive noise problem of medical image noise reduction technology has been noticed by many researchers. At the same time, doctors often need to rely on clinical experience and academic background knowledge in the follow-up diagnosis of lesions, so it is difficult to promote medical clinical diagnosis technology. Therefore, research into medical image denoising technology and computer-aided diagnosis emerges in an endless stream because of the demand for medical treatment.

Medical physicists and radiologists began to focus on computer-aided detection and diagnosis in the mid-1980s [11]. The results of computer-aided diagnosis have a significant influence on doctors’ judgment of the lesion body, and the merits and demerits of medical image noise reduction technology are also of great importance to the results of computer-aided diagnosis. With the increasing precision of cancer medical images, these images contribute a mass of useful information. To use this image information accurately and efficiently, the research into computer-aided diagnosis based on cancer medical images has become a hot topic in the industry. Accurate diagnosis or evaluation of disease in the field of medical imaging relies on image interpretation and acquisition. The interpretation of medical images is conducted mainly by doctors who are affected by subjective influence. Many diagnostic tasks require detecting anomalies through an initial search process and quantifying changes in measurements and time. Computerized tools such as machine learning and picture analysis play a pivotal role in enhancing diagnosis, sifting through areas that need treatment to help specialists do their workflow. In the development of research, the neural network has been extensively used to research intelligent methods to assist medical image diagnosis and has made significant progress. Deep learning quickly proved to have fundamental abilities to improve accuracy, and it has also broken new ground of data analysis that is evolving at an unprecedented rate.

**Figure 1:**
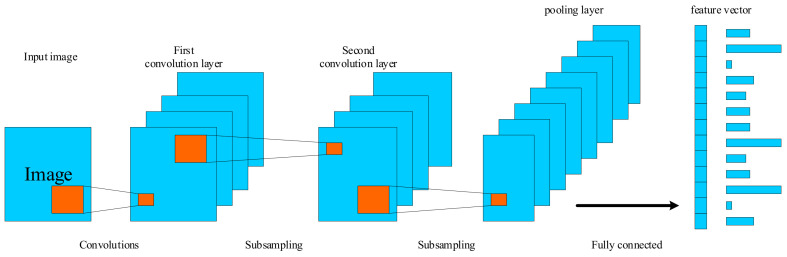
The process of CNN.

A convolutional neural network (CNN) is a feedforward neural network that usually consists of one or more convolutional layers and fully connected layers and includes ReLU and pooling layers [12]. The typical CNN for processing images consists of convolutional filter layers mixed with pooling or data compression layers. The general process of CNN [13] is shown in Figure 1. A convolution filter processes a small piece of the input image. Similar to the human brain’s low-level pixel processing, convolution filters can detect highly relevant image features. The output of a CNN is usually the label of one or more probabilities or categories corresponding to images. Convolution filters can learn directly from the trained data, which reduces the need for time-consuming manual labeling of features. Without the assistance of convolution filters, in the stage of image preprocessing, filters designed for specific applications and some features to be calculated cannot be separated from manual methods. Many studies have found that the computer-aided diagnosis method based on neural networks has a better effect than the traditional method in tumor segmentation and classification, which indicates that the neural network-based method has a broad research space and has a better clinical application prospect [14].

In this article, we will present the background of tumor research, introduce the traditional methods of computer-aided diagnosis of tumors, and introduce the segmentation and classification of tumor images. We will also demonstrate diagnostic methods based on convolutional neural networks. Finally, some recent advances in medical tumor image processing and classification detection using convolutional neural networks will be discussed. In this article, we discuss how CNNs can help doctors detect tumors, summarize the comprehensive information advantages of this paper, and provide a reference for the future development of CNN-based computer-aided tumor detection systems.

## 2. Background

### 2.1. Symptoms of Tumors

Many of the different types of cancer that develop from primary tumors are hard to detect in their early stages, generally are not detected until late, and often miss the best time for treatment. Therefore, the comprehensive early detection of cancer is particularly important. Diagnostic tests for tumors usually detect the vast majority of possible early cancers on the micron scale. Early diagnosis can kill cancer in time so that the patient does not have to suffer the pain and life risk caused by the spread and progression of cancer.

According to the degree of differentiation of tumor cells, soft tissue sarcomas can be divided into four histological subtypes: highly differentiated, moderately differentiated, poorly differentiated, and undifferentiated [15]. The degree of differentiation is the lowest, and the degree of malignancy is higher. The degree of tumor differentiation is not uniform, and physicians have certain subjective factors in judging the situation of a tumor. In general, the early symptoms of the tumor are related to the type and location of the tumor. The tumor’s growth at the primary lesion site leads to changes in the anatomical structure and histological morphology at the lesion site, leading to corresponding changes in symptoms. The formation of tumors in the tissue and the relationship between tumor and adjacent tissue can be used as the basis of tumor examination and diagnosis. For example, some benign tumors may have no apparent symptoms in the early stages. A malignant tumor is related to the specific growth site of the patient. If the malignant tumor grows in a certain site, the patient may only have local swelling or pain in the early stage. As a result, each malignant tumor has different growth sites and different symptoms. Because the clinical manifestations of benign and malignant tumors are different, the early systemic symptoms of tumors are generally mild and limited. If symptoms are detected early, it can help doctors recommend appropriate treatment in time. However, if the tumor is ignored early on, it can lead to follow-up treatment and gradually develop into cancer [16].

Therefore, early and accurate diagnosis can help doctors make the correct diagnosis and treatment plan for the patient’s disease in a timely manner, which is particularly important and can also reduce the mortality rate of cancer patients.

### 2.2. Background of Medical Facilities

Medical imaging technology is an important branch of modern medicine and has been widely used in clinical treatment, especially in diagnosing tumors.

Common medical imaging techniques include digital X-ray radiography (X-ray), Ultrasound Color Doppler (UCD), computed tomography (CT), and magnetic resonance imaging (MRI). Doctors can get a quick and detailed picture of a patient’s condition by taking and imaging the affected organ and making a more accurate diagnosis. Developing better treatment plans can also (i) reduce the rate of misdiagnosis effectively, (ii) promote the efficiency of the entire medical system, and (iii) alleviate the suffering of tumor patients.

Medical imaging technology is the most direct bridge between doctors and patients to communicate their conditions. Among all kinds of high-precision medical equipment in hospitals, medical X-ray diagnostic equipment is the earliest and most widely used means of medical imaging examination. X-ray images have apparent advantages in the examination of dynamic and subtle lesions. Although other medical imaging devices have emerged recently, X-ray diagnostic devices still maintain irreplaceable advantages in bone, gastrointestinal, vascular, and breast examinations. However, the X-ray image is the information carrier of X-ray radiation, so it will inevitably harm the human body. Therefore, the speed of MRI, and the low risk of human injury, make the technology widely used in clinical diagnosis. Scientific research applications have dramatically advanced fast medical developments.

In X-ray imaging mode, the X-ray detection of breast and lung cancer cells may be affected by the density of body tissues. In UCD, the resonance of sound waves can be used to detect the texture and density of body tissue. These images usually show the shape of an organ or a tumor. However, UCD usually has poor image quality, and it is always difficult to obtain a precise boundary of the cancer area and identify small nodules. CT can take a suite of images showing horizontal slippage in the area. The quality of the image shows the shape and density of the organ tissue. These imaging devices are combined with computer science to produce computer-aided diagnostic systems. It is a computer program for pathological diagnosis that helps detect and calculate tumor lesions by combining techniques for processing medical images and other possible biochemical and physiological means with image analysis. Computer-aided diagnostic systems used for tumor detection usually record medical images using appropriate imaging systems. The captured image is then used through various software-based algorithms to separate characteristic tumor areas from the rest of the picture. Features of biomedical knowledge, such as shapes and texture, can be extracted and form a feature space that describes the biometric features of areas of possible variation.

The segmentation of images plays a vital role in computer-aided diagnosis systems. It aims to isolate feature regions from the rest of the picture. It can also combine visual features such as texture information with other biological features to differentiate between different areas of a picture. Thus, unaffected areas can be automatically screened out, and suspicious areas similar to pathological features such as irregular texture are left behind. Determining the location and size of the tumor and accurate segmentation results are essential for treatment planning. Among different medical imaging methods, image segmentation is a very effective way to detect cancer. However, owing to the differences in biological information in different positions of the human anatomy, it is almost inevitable that human intervention is required. The initial conditions for setting classification or training data need to be provided by an experienced clinician. However, there have been numerous studies to detect various types of cancer based on information extracted from medical images. However, most image-based tumor diagnostic systems detect only a onefold mold of the tumor. Most image processing algorithms process data from the same pattern or a specific set of images. Image processing extensions can combine pivotal features extracted from different modes of tumor images to improve feature detection, such as tumor location and shape. The advantages, disadvantages and applications of tumor detection methods are shown in Table 1.

### 2.3. Diagnostic Methods Based on Convolutional Neural Network

In the medical field, computer-aided diagnosis helps clinicians to transform subjective image information into objective image information to assist clinical decision-making. However, deep learning based on a convolutional neural network (CNN) has obvious advantages over traditional computer-aided diagnosis. It has a simpler extraction process, can automatically extract distinctive feature information from data sets, and its performance is more systematic and easier to adjust. As machine learning and deep data mining make cancer detection easier, researchers can extract characteristic information from the data, which can be used to predict cancer.

Esteva et al. [17] used image pixels and disease labels as inputs for end-to-end training to classify skin tumors through a single convolutional neural network. The dataset for training CNN was 129,450 clinical images. By identifying keratinocyte carcinoma and benign seborrheic keratosis, the most common cancer, and malignant melanoma and benign nevus, the deadliest skin cancer, and comparing it with the diagnosis of professional dermatologists, CNN demonstrated a level of competence comparable to that of dermatologists. Vivanti et al. [18] proposed automatic liver tumor delineation based on a robust CNN approach in longitudinal CT studies for patient-specific and global CNN training on a tiny contour image dataset. Different from other deep learning methods of medical image processing, which require a huge number of annotated training data sets, the way proposed by the authors utilizes a subsequent frame structure to generate accurate tumor tracking through tiny training data sets, thus reducing the problem of manual processing to a certain extent. Khosravi et al. [19] constructed an independent pipeline containing several CNN-based computational methods to effectively classify two lung cancer subtypes, four bladder cancer biomarkers, and five breast cancer biomarkers. The pipeline classification includes three training strategies, CNN, Google’s Inception, as well as Inception and ResNet algorithms. In a wide range of tumor heterogeneity, the proposed method achieved an accuracy of 100% in differentiating various cancer tissues, 92% in subtypes, 95% in biomarkers, and 69% in scores.

## 3. Traditional Computer-Aided Tumor Diagnosis

**Figure 2:**
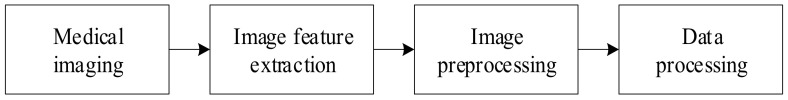
The process of image classification.

Computer-aided diagnosis has always been the focus of medical research. Many computer-aided diagnosis technologies for different pathological images continue to appear and develop rapidly, which plays a positive role in assisting doctors in diagnosing diseases to a great extent. The process of computer-aided diagnosis, as shown in Figure 2, consists of firstly inputting and segmenting medical images to obtain several segmentation results of the same region of target, then the results of the feature segmentation are extracted to feature pool formation, and the first feature subset is extracted from the feature pool. Among them, the results of the segment images are obtained by adopting several complementary segmentation methods for the same target region. A systematic literature search was conducted in the following electronic databases, all without any language restrictions: PubMed via Medline, EMBASE. The search terms are common names, scientific names and synonyms of “Image feature Extraction “, “Feature reduction“, “Traditional image classification“. There is no limit to the search function by manually searching references for papers.

In computer-aided diagnosis system design, the calculation can improve the effectiveness of the feature information in the feature subset, reduce the rate of false-positive detection in the classifier, and enhance the accuracy of the diagnostic results.

### 3.1. Feature Extraction

In recent years, image processing technology has developed rapidly and has been more and more widely used. Some of these technologies are pretty mature and have yielded excellent benefits. Research on new processing methods is the main task of image processing to develop a broader range of applications. Traditional digital image processing advantages are high accuracy, rich content processing, flexible processing, and complex non-linear processing. The disadvantage is that the processing speed is slow for complex processing. Processing methods of the digital image can be divided into two categories: spatial and transform domain methods. The spatial domain approach treats the picture as a set of pixels on a plane and then deals directly with two-dimensional functions. The method of transforming the domain is to transform the orthogonality of the image first. Then, the array of coefficients in the transform field is retrieved, and various processing is performed. After processing, the inverse transformation of the space domain is carried out to obtain the processing result. This type of processing includes filtering, data compression, feature extraction, and other processing.

Low-level feature extraction mainly focuses on texture, color, local features, shape features, and so on. Commonly used feature extraction methods include scale-invariant feature transformation, accelerated robust feature extraction, fast orientation and rotation simplification, gradient direction histogram, etc.

#### 3.1.1. Scale Invariant Feature Transform

A local feature descriptor, Scale Invariant Feature Transform [20] (SIFT), proposed by Lowe in 1999, was improved in 2004 [21]. The image after the SIFT algorithm is represented as a set of 128-dimensional feature vectors because the SIFT operator uses 128-dimensional feature vectors to describe the feature points detected in the picture. The feature vector set is invariant to image scaling and translation and is an excellent local feature description algorithm. The process of SIFT algorithm is pole detection in scale space, accurate location of key points, orientation determination of key points, and generation of feature vectors. The detection of feature points needs to know the location and scale of feature points, and the feature points to seek are to find the points whose position does not change in continuous scale space.

The Gaussian function is the core of scale space. Assuming that the Gaussian function of a variable scale space is $G\left(x,y,\sigma \right)$, the scale space of the original image $I\left(x,y\right)$ can be defined as:

(1) $$L\left(x,y,\sigma \right)=G\left(x,y,\sigma \right)\;*\;I\left(x,y\right)$$

where the convolution operation is expressed as ∗. A higher σ express the fuzzy general picture of the image, whereas a lower σ represents the precise detail of the image. The Gaussian function $G\left(x,y,\sigma \right)$ is defined as:

(2) $$G\left(x,y,\sigma \right)=\frac{1}{2\pi \sigma^{2}}e^{-\left(x^{2}+y^{2}\right)/2\sigma^{2}}$$

**Figure 3:**
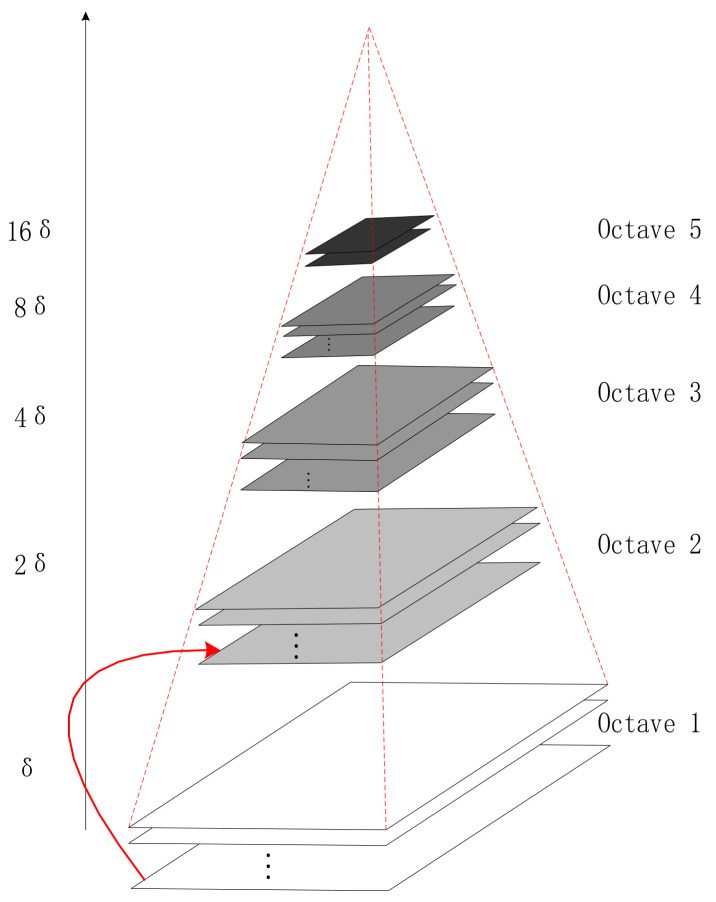
Gaussian pyramid.

Finally, the Gaussian pyramid (GP) is obtained through a suite of scale-space transformations and second sub-sampling, as shown in Figure 3.

The scale of space is realized through the GP [22]. The pyramid model of a picture means a tower-like model that continuously reduces the order of the original image and obtains a suite of images of different sizes, from small to large and from top to bottom. Each pyramid has a total of n layers. The first layer of the golden tower is the original image, and each downward sampling results in a new image of a layer of the pyramid. The size of the top image and the original image determines the number of layers of the pyramid.

On the basis of simple down-sampling, a Gaussian filter is added to the Gaussian pyramid. Each layer of the pyramid contains multiple Gaussian blur images, which are collectively called octaves in each layer of the pyramid. Using the difference of Gaussians (DOG) pyramid instead of the Gaussian pyramid in extremum detection can save plenty of computation time. The next step in constructing the differential GP is to find the extreme point in DOG, which is the candidate point of the feature point. The extremum point is also the candidate point of the key point, comprised of the DOG space local extremum points. The key points were initially explored by comparing two adjacent layers of images of each DOG in a group. The search for the DOG function extreme point requires that each pixel be compared to all its adjacent points, whether smaller or larger than its adjacent points in the scale and image domain. In scale space, the SIFT algorithm uses DOG function $D\left(x,y,\sigma \right)$ to find stable and invariant extreme points:

(3) $$D\left(x,y,\sigma \right)=\left[G\left(x,y,k\sigma \right)-G\left(x,y,\sigma \right)\right]\;*\;I\left(x,y\right)=L\left(x,y,k\sigma \right)-L\left(x,y,\sigma \right)$$

where kσ and σ are the smooth scales of two continuous images. The Laplacian of Gaussian (LOG) operator can detect the speckle feature of the image at different scales to detect the fixed point of the picture under the change of the mesoscale, but the efficiency of LOG operation is not high. DOG is an approximation of LOG, and the relationship is as follows:

(4) $$\sigma \nabla^{2}G=\frac{\partial G}{\partial \sigma }\approx \frac{G\left(x,y,k\sigma \right)-G\left(x,y,\sigma \right)}{k\sigma -\sigma }$$

Therefore, we have:

(5) $$G\left(x,y,k\sigma \right)-G\left(x,y,\sigma \right)\approx \left(k-1\right)\sigma^{2}\nabla^{2}G$$

where $\sigma^{2}\nabla^{2}G$ is the expression of the scale normalization operator. In all scales, k−1 is a constant, and when k approaches 1, the error approaches 0. However, this error does not affect the position detection of the extreme value.

However, the extrema generated in this way are not all stable feature points because some extrema have weak contrast, and the DOG operator will generate a strong edge response. In the key point localization step, the position and scale of the key point can be accurately determined by fitting the quadratic function in three-dimensional space with the selected extremum points. In addition, the feature points of SIFT can be screened out by deleting the extreme points with weak contrast and the edge response points. The SIFT algorithm benefits feature stability, but this method has some drawbacks, such as poor real-time performance and a weak ability to extract feature points from targets with smooth edges. The proposed Speeded Up Robust Features improved the extraction and description of the features and completed the extraction and description of the features more efficiently.

#### 3.1.2. Oriented Fast and Rotated Brief

The FAST (Features from Accelerated Segment Test) algorithm was adopted for feature point detection by Oriented Fast and Rotated Brief (ORB) [23]. To find those outstanding points is the core idea of FAST, which means taking a point and comparing it with the points around it. It can be thought of as a characteristic point if most of the points are different from it. The calculation of FAST first selects a pixel point P from the picture and then determines whether it is a characteristic point. Its grayscale value is set to $I_{p}$ and an appropriate threshold value T is set, which means that when the absolute value of the difference between two points of grayscale value is greater than T, the two points are considered to be different. A corner is n consecutive points that are different from P. This method can quickly exclude vast non-feature points.

Feature descriptors of feature points are derived from the feature points calculated by FAST, and then the attributes of these feature points are described in some way. ORB uses the BRIEF algorithm to calculate feature point descriptors. Its core idea is to select the comparison results of N point pairs around the key point P as the descriptors.

**Figure 4:**
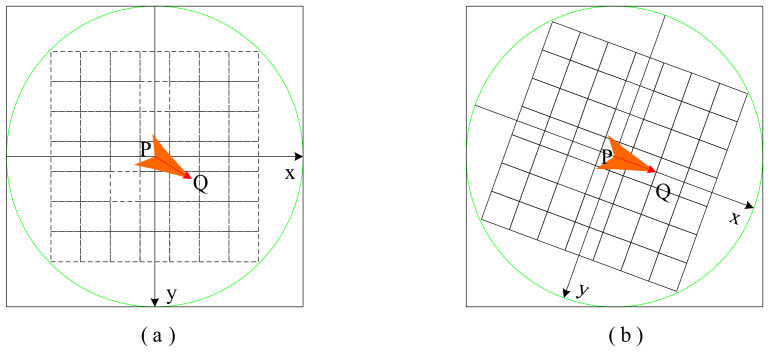
The coordinate system is created when calculating the BRIEF descriptor. (**a**) is the selected point pair, and (**b**) is the matching point pair calculated after rotation.

In Figure 4, P is the key point, and Q is the point of the selected region. Inside the circle is the region of picking points, and each small grid represents a pixel. The central area of the circle is regarded as a board, and the mass of each point on the board is equal to its corresponding pixel value. The same feature point should have sufficiently similar descriptors, which is called the reproducibility of descriptors. The descriptors obtained by the BRIEF algorithm do not have sufficiently similar descriptors with the same feature points in images with different sizes, directions, and shades, so descriptors have no robustness. The ORB does not address scale consistency, but BRIEF descriptors do not have rotation invariance. Figure 4a represents the selected point pairs, and Figure 4b represents the matching point pairs calculated with the coordinate system in the main direction by rotation of a certain Angle. Although rotation invariance is added to the improvement of BRIEF, at the same time the discriminability of feature descriptors is reduced. The most important characteristic of the ORB algorithm is its high calculating speed, which first benefits from using FAST detection characteristic points. Then, the BRIEF algorithm is used to compute the descriptor, whose unique representation of a binary string not only has space-saving storage but also minimizes the matching time.

### 3.2. Feature Reduction

In practical application, the probability distribution estimation is inaccurate owing to the limited number of training samples or the mismatch between the assumed probability model and the real model. After the quantity of features increases to a certain critical point, the further increase will deteriorate classifier performance. For high-dimensional data, the dimension of the feature vector makes pattern recognition difficult to solve. In this case, it is usually required to reduce the dimensionality of the eigenvector first. Feature vectors often contain redundant information. Some features may have nothing to do with classification problems, and there is a strong correlation between features. Therefore, dimension reduction of the eigenvector is feasible. Usually, methods to reduce the feature vector dimension can be applied to feature combination or feature selection. Different training algorithms for different training objectives include linear discriminant analysis [24] and principal component analysis [25].

Feature dimension reduction directly reduces the dimension of the training model after feature selection, but the feature matrix with large computation and long training time also needs to be reduced. The commonly used dimensionality reduction methods are linear discrimination analysis and principal component analysis. The low-dimensional sample space that maps the original data is the essence of both methods. However, the methods used are different. Principal component analysis aims to make the mapped samples have greater divergence, whereas linear discriminant analysis aims to make the mapped samples have the best classification performance.

Principal component analysis (PCA) is a commonly used unsupervised linear dimension reduction algorithm [26]. This algorithm aims to retain more characteristics of the original data points while using fewer data dimensions. Usually, mapping all the points together reduces the dimension, and almost all the information is lost. However, if the mapped data have a large variance, it can be considered that the data points will be more scattered, thus retaining more information. Therefore, in PCA dimension reduction, the loss of original data information is minimal.

Different from PCA, which does not consider the output of sample category, each sample of the data set in linear discriminant analysis (LDA) is output by category [27]. LDA can be understood as the maximum inter-class variance and the minimum intra-class variance after projection. There are many similarities between PCA and LDA. Both assume that the data conform to the Gaussian distribution and adopt the idea of matrix eigendecomposition in dimensionality reduction. Compared with PCA, the dimension reduction of LDA can be lessened to the dimension of class number K−1, whereas PCA has no limit. The advantage of LDA is that it can use prior-like knowledge experience to reduce dimension, whereas PCA of unsupervised learning cannot use prior-like knowledge. The variance-dependent sample classification information may have the problem of overfitting data, and the effect of dimension reduction is not good. On the contrary, it is superior to PCA and other algorithms.

### 3.3. Classification

The categorizer can classify the image described by a vector of a fixed dimension. The most frequently used methods include support vector machine (SVM), *k*-nearest neighbor classifier, Bayes classifier, and other classifiers. SVM is the widest categorizer, and the kernel method has good performance in the task of the traditional classification images.

#### 3.3.1. Support Vector Machine

**Figure 5:**
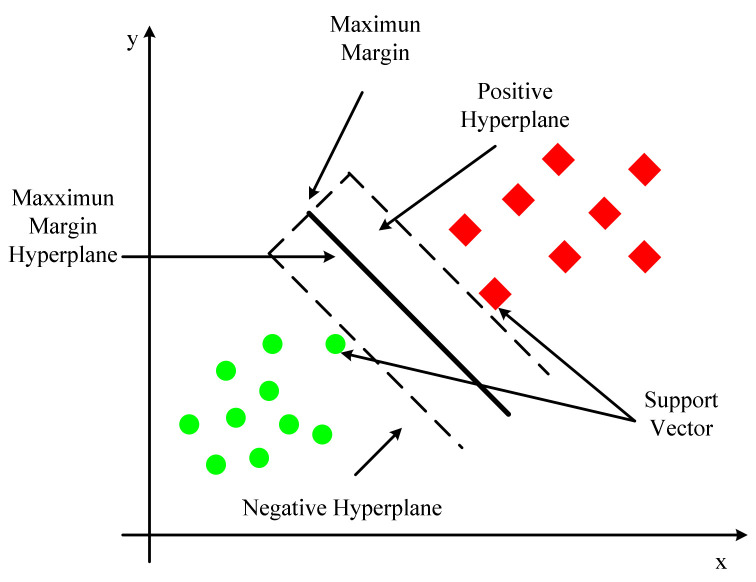
Support vector machine.

In conventional machine learning, the support vector machine (SVM) [28] is an important classification algorithm. As shown in Figure 5, the model assigns instances’ eigenvectors to specific locations in space. The goal of the SVM is to build a line that distinguishes between the two types of points so that the line can be used to classify additional points in the future. SVM is appropriate for nonlinear, high-dimensional classification problems with small and medium-sized data samples. SVM was nearly universally regarded as the most successful and best-performing machine learning algorithm prior to the emergence of deep learning. The dividing line in higher dimensional space similar to that in two-dimensional space is called a hyperplane. On the hyperplane, both sorts of sample points have a lot of points. These locations are known as support vectors because they play a role in defining the hyperplane’s segmentation.

The objective function of SVM first assumes that the hyperplane is $w^{T}x+b=0$. The distance from point x to the hyperplane is represented by $w^{T}x+b$ given a sample x. The classification is correct by observing whether $w^{T}x+b$ and y are equal. The function interval is defined as $\gamma^{\prime }$:

(6) $$\gamma^{\prime }=y*\left(w^{T}x+b\right)$$

In most cases, the function interval does not reflect the distance between the point and the hyperplane. The function interval will also expand by the corresponding multiple when w and b are scaled up equally. Therefore, geometric intervals are introduced. The normalized constraint on w in the denominator is defined as:

(7) $$Y=\frac{y*\left(w^{T}x+b\right)}{||w_{2}||}$$

The geometric interval is the distance from the point to the hyperplane in higher dimensional space, which can truly reflect the distance between the point and the hyperplane.

The geometric interval between the support vector and the hyperplane is maximized according to the SVM principle. Hence, the objective function may be represented as:

(8) $$\mathrm{Max}Y=\frac{y*\left(w^{T}x+b\right)}{||w_{2}||}$$

(9) $$s.t.\;y_{i}\left(w^{T}x_{i}+b\right)\geq 1\left(i=1,2,\ldots ,m\right)$$

The enlargement or compression of the molecule (the function interval from the support vector to the hyperplane) is equal to 1. Then the goal function can then be changed into:

(10) $$\mathrm{Max}Y=\frac{1}{||w_{2}||}$$

(11) $$s.t.\;y_{i}\left(w^{T}x_{i}+b\right)\geq 1\left(i=1,2,\ldots ,m\right)$$

The SVM algorithm can map to a high-dimensional space using the kernel function, and the kernel function can solve nonlinear classification, maximize the distance between the classification target sample and the decision surface, and provide a superior classification effect than other approaches. However, large-scale training samples are difficult to execute, and it is not ideal for multiple classification tasks.

#### 3.3.2. *k*-Nearest Neighbors Algorithm

**Figure 6:**
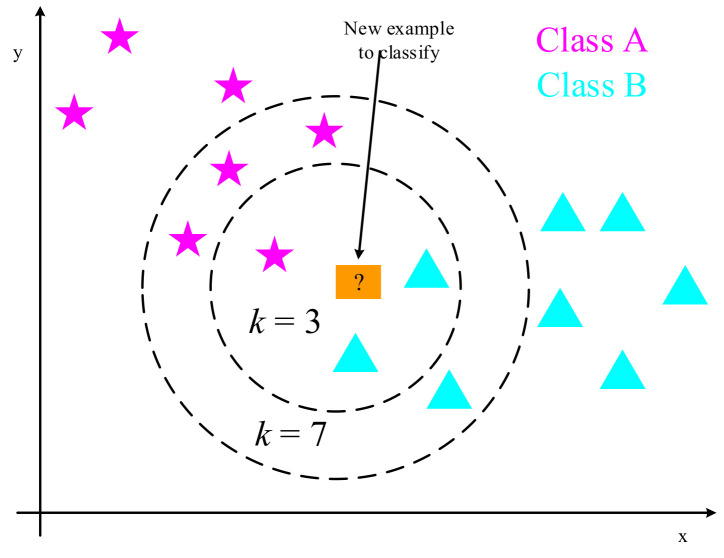
*k*-nearest neighbors algorithm.

A simple classification and regression method is the *k*-nearest neighbors algorithm (kNN) [29,30,31]. As shown in Figure 6, it works by straightening a two-dimensional vector into a one-dimensional one and judging the similarity between the vectors based on the distance measure. To put it another way, given a training data set, the k examples closest to a new input instance are located in the training data set. The input instance is classified into this class, as are the majority of these k instances. This simple method without feature extraction loses the information of the most important adjacent pixels in the two-dimensional vector. Choosing the value of k is one of the most important aspects of the *k*-nearest neighbors method. If a smaller value of k is selected in the experiment, it means that the overall model will become complex. Some noises are learned into the model, whereas the real distribution of data is ignored, and overfitting can occur easily. Choosing a bigger value of k in the experiment, on the other hand, is similar to making a prediction using training data from a broader neighborhood. In this circumstance, the training instance, which is located far from the input instance, will also play a part in the forecast, causing the prediction to be incorrect. When the value of k is increased, the entire model becomes simpler. Oversimplified models tend to ignore a lot of helpful information in training data instances. When a smaller number is chosen, the cross-validation approach is commonly used to determine the best k value, and a better result can be obtained based on experimental parameter tuning.

Assume that the eigenspace X is a real vector space $R^{n}$ of n dimensions, where $x_{i,}x_{j}\in X$, $x_{i}={\left(x_{i}^{\left(1\right)},x_{i}^{\left(2\right)},\ldots x_{i}^{\left(n\right)}\right)}^{T}$, $x_{j}={\left(x_{j}^{\left(1\right)},x_{j}^{\left(2\right)},\ldots x_{j}^{\left(n\right)}\right)}^{T}$. The $L_{p}$ distance of $x_{i},\;x_{j}$ is defined as:

(12) $$L_{p}\left(x_{i},\;x_{j}\right)={\left(\overset{n}{\underset{l=1}{\sum }}{\left|x_{i}^{\left(l\right)}-x_{j}^{\left(l\right)}\right|}^{p}\right)}^{\frac{1}{p}}$$

Here p≥1. We have Euclidean distance when p=2:

(13) $$L_{2}\left(x_{i},\;x_{j}\right)={\left(\overset{n}{\underset{l=1}{\sum }}{\left|x_{i}^{\left(l\right)}-x_{j}^{\left(l\right)}\right|}^{2}\right)}^{\frac{1}{2}}$$

We have Manhattan distance when p=1:

(14) $$L_{1}\left(x_{i},\;x_{j}\right)=\overset{n}{\underset{l=1}{\sum }}\left|x_{i}^{\left(l\right)}-x_{j}^{\left(l\right)}\right|$$

We obtain the maximum distance at each coordinate when p=∞:

(15) $$L_{\infty }\left(x_{i},\;x_{j}\right)=\underset{l}{\max}\left|x_{i}^{\left(l\right)}-x_{j}^{\left(l\right)}\right|$$

### 3.4. Disadvantages of Traditional Methods

Candidate lesion locations are typically found by monitoring systems or traditional image processing techniques in regular computer-aided tumor diagnosis approaches. The process of determining the location of a lesion is done in stages and is usually marked by a significant number of hand-crafted features. To detect the likelihood of genuine lesions, the classifier is utilized to map eigenvectors to candidates. As a result, computer-aided diagnosis is an area of medical picture analysis that can be enhanced [10]. According to the pixel data of the image, the traditional algorithm uses the designed formula to detect the features of the image, including corner, contour, color gradient, and others. Different algorithms have different accuracy in detecting these features, and different experimental methods also have their feature extraction methods. When the same image has some linear or nonlinear transformation, it will cause some interference in feature extraction, such as scaling, rotation, translation, affine transformation, or deformation. Therefore, modern and more advanced convolutional neural network algorithms are superior to older algorithms in certain aspects or overall accuracy. Different algorithms have different abilities to deal with these transformations, and the stronger the problem-solving ability, the better the robustness.

## 4. Basic Knowledge of Convolutional Neural Networks

### 4.1. Basic Principles of Convolutional Neural Network

The appearance of the neural network, in comparison to the prior computer-aided diagnosis system, makes picture feature processing involve less engineering. A deep feedforward convolutional neural network differs from a standard neural network with fully connected layers in that it has a significant generalization ability [32].

Hubel and Wiesel’s [33] investigation of the visual system in the cat brain in 1962 led to the construction of the first CNNs. They carefully generated a map of the visual cortex by recording the electrical activity of individual neurons in the cat’s brain. In 1980, a Japanese scientist named Kunihiko Fukushima proposed a neural network layout that included a convolutional layer and a pooling layer. [34]. Yann LeCun proposed Lenet-5 in 1998, based on the earlier one, and added the backpropagation algorithm to the training of the neural network structure, which is the prototype of today’s CNN [35]. Although the original CNN is excellent in applications such as number recognition, it falls short of algorithms such as the support vector machine in training problems and general practical tasks, and as a result, it has been overlooked. Until 2012, Hinton’s team offered AlexNet [36] in the ImageNet image recognition challenge, which introduced a novel deep structure and dropout mechanism, lowering the error rate and refocusing CNN development in the field of image recognition. Subsequent combinations of studies gradually reduced the error rate to an infinite level of human recognition by subtly modifying the structure of the neural network and adding different layers. In a few short years, the development of the CNN has occupied the center of gravity in the field of computer science. The blossoming of CNNs has led to many changes in other fields, including the field of medicine. CNN has gradually replaced the traditional neural network method. During image processing, the traditional neural network method can easily slow down the model processing speed due to a large amount of parameter calculation, whereas CNN can reduce the dimension of images with large amounts of data, effectively retain image features, reduce feature quantities, and speed up model calculation. Therefore, traditional neural networks are inferior to CNNs in terms of computing resources and parameter tuning. A systematic literature search was conducted in the following electronic databases: PubMed via Medline, EMBASE. The search words are "Convolutional Neural Networks", "Pooling layer", "activation function", "Propagation", "Training Neural network", and” network architecture”. All databases have no language restrictions.

### 4.2. The Basic Structure of the Convolutional Neural Network

The basic structure of the convolutional neural network is composed of an input layer, a convolutional layer, a pooling layer, a fully connected layer, and an output layer. The convolutional layer is usually responsible for extracting local features in the image. The pooling layer is used to greatly reduce the parameter magnitude equivalent to feature selection. The full connection layer is similar to the part of the traditional neural network, which classifies the data and outputs the desired results.

The image’s pixel value is preserved in the input layer. The output of the neuron connected to the input local region is decided by the scalar product of the region related to the input volume via weight computation in the convolutional layer. The rectified linear unit’s job is to take the activated output from the preceding layer and apply an activation function to it. After that, the pooling layer is employed to reduce the number of parameters in this activation by subsampling along the input’s spatial dimension. The fully connected layer will perform similar tasks to the standard ANN and generate category scores from the activation for classification. To boost performance, ReLU is recommended to be utilized between layers. Using this simple transformation method, CNN may employ convolution and subsampling techniques to modify the original input picture data layer by layer and obtain classification scores for classification and regression.

However, in terms of model creation and optimization of CNN architecture, it still takes quite a long time to perfect the hyperparameters and connectivity of each layer, and simple CNN architecture is not enough to meet the real usage requirements.

#### 4.2.1. Convolutional Layer

**Figure 7:**
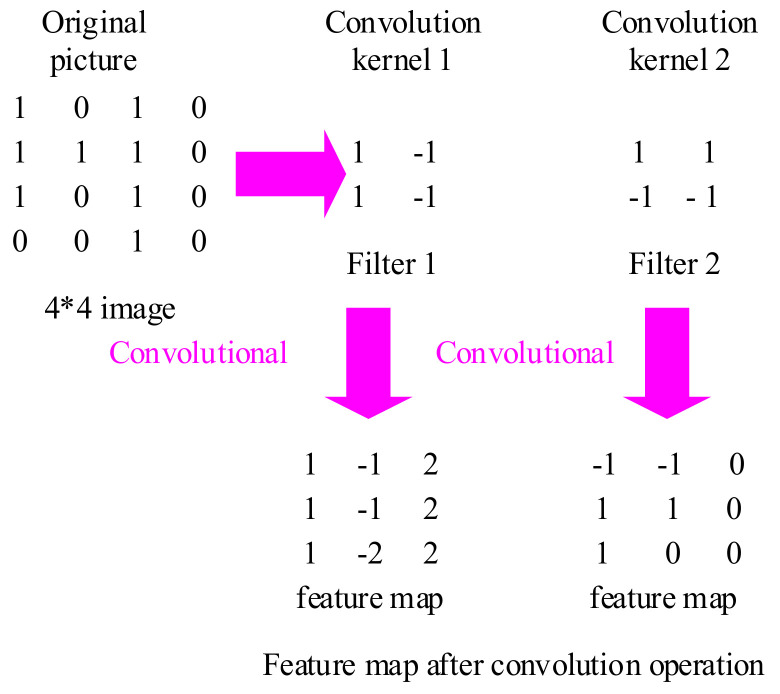
Image feature convolution process (Where 4*4 is the size of image).

In a convolutional neural network, the convolutional layer plays a significant function, and the learning kernel is the focus of the layer’s parameter. The convolution kernel is modest in spatial dimension and dispersed throughout the entire input depth. The convolution layer is made up of a series of filters, which can be deemed a two-dimensional digital matrix [37]. The convolution kernel can be used to identify a typical feature of the image, filter each small area in the image, and get the eigenvalues of these small areas, as shown in Figure 7. When picture data enters a convolution layer, it convolves each filter across the input’s spatial dimension to form a 2D activation map. When sliding input, these activation maps can be viewed, and scalar products can be computed for each value in the core. The network will activate and learn the kernel from the input image when it searches for a specific feature in a given spatial position. Each kernel has an activation map that is layered to the depth dimension to produce the convolution layer’s whole output volume.

In traditional artificial neural networks, training neural networks on the input of image equal pixels usually results in models too large to be trained effectively. The convolution layer exists to alleviate data processing strain, and each convolution layer neuron is only connected to a small portion of the input volume. The convolution kernel can be shared among neurons in the same layer, making higher-order data processing very straightforward. In addition, after using the convolution kernel, the size of the image becomes smaller, which is convenient for subsequent calculation. The convolution layer can considerably reduce the model’s complexity by tweaking hyperparameters like depth and stride length. In general, the automatic training of the system can be completed only by designing the size, number, and sliding step of the convolution kernel, and manual selection of features is not required. The size and number of each convolution kernel can be defined by itself. Fewer convolution kernels are set for the convolutional layer closest to the input layer, and more convolution kernels are set for the later convolutional layer.

#### 4.2.2. Pooling Layer

The pooling layer can compress the data dimension more effectively than the convolutional layer, which not only reduces the amount of computation but also successfully avoids overfitting [38]. In the same way that adjacent pixels in an image have similar values, adjacent output pixels in a convolutional layer have similar values. As a result, the majority of the data in the convolutional layer output is redundant. For the convolutional layer, it will change the dimensions of the original pixel matrix from three dimensions, namely, height, width, and depth, which are determined by the number of convolutional cores. For the pooling layer, the pixel matrix is only changed in the direction of height and width, and there is no change in the direction of depth. The parameters of the convolution kernel are affected by the backpropagation and will change each iteration. For the pooled layer, there is no parameter adjustment during iteration. At the same time, since the pixel value is generally large for the edge of the object, the key information can be retained through maximum pooling. By lowering the size of the input, the pooling layer addresses the strong edge detection problem and minimizes the number of output values. As a result, most pooling procedures are simply average, minimum, or maximum operations.

**Figure 8:**
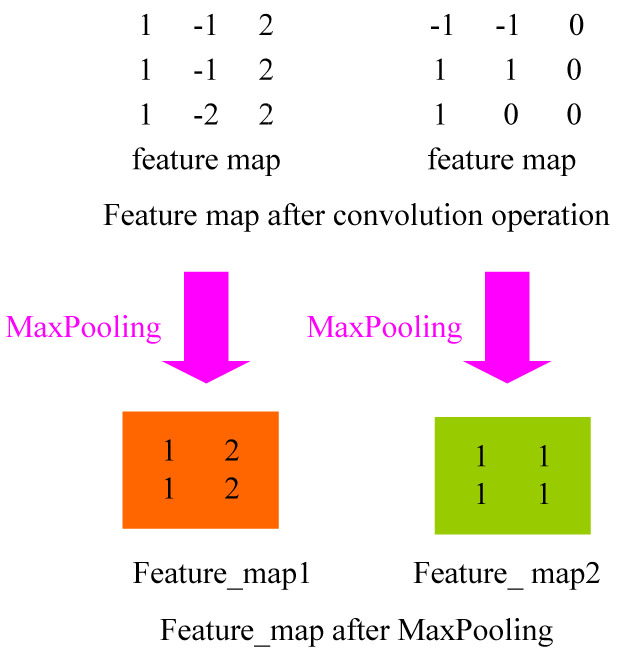
Image feature maximum pooling process.

Common pooling layers include max-pooling [39,40], average pooling [41], random pooling [42], and global average pooling [43]. The average value of the image region is calculated as the region’s pooled value using average pooling. The value of the mistaken item in the lower layer will be evenly distributed among all neurons in the corresponding block in the higher layer when average pooling is used. The maximum pooling method involves choosing the picture region’s maximum value as the region’s pooled value. The error item value of the lower layer will be transported unchanged to the neuron corresponding to the maximum value in the corresponding block of the higher layer during max-pooling, but the error item value of other neurons will be 0. The error in feature extraction is mostly caused by an increase in the variance of the estimated value due to the size of the neighborhood and a departure from the estimated mean value due to the convolutional layer’s parameter error. In general, average pooling can reduce the variance increase in the estimated value caused by the neighborhood size limitation, while retaining the majority of the image’s background information. The variation of the estimated mean value, which is primarily responsible for retaining texture information, is caused by the parameter error of the maximum pooling reduction convolutional layer. Random pooling is between the former two. By assigning a probability to pixels according to their numerical size, sub-sampling is carried out according to the probability. In terms of the sample average, it is approximate to average pooling. Locally, however, it is subject to the maximal pooling criterion. The pooling process is shown in Figure 8.

From a computational standpoint, the largest pool is the simplest. Consider max-pooling: Each convolution kernel may be considered a feature extractor, with different convolution kernels handling different feature extraction. It assumes that the first convolution kernels’ design can extract the vertical direction’s characteristics, whereas the second convolution kernels’ design can extract the horizontal direction’s features. After max-pooling, the extracted feature values can be identified. The remaining values are discarded to lower the size of the feature graph in the subsequent calculation, thus reducing the parameters and the amount of calculation.

#### 4.2.3. Full Connection Layer

**Figure 9:**
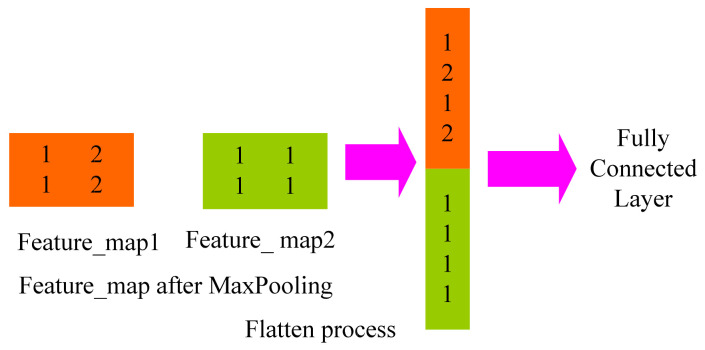
Image feature “flatten” layer.

Each full connection layer is a tiled structure made up of many neurons [44]. To obtain the ultimate desired result, the data processed by the convolution layer and the pooling layer are fed into the complete connection layer. Only once the convolutional layer and the pooling layer have reduced the dimensionality of the data can the entire connection layer be shielded from the effects of big data volumes, high computational costs, and low efficiency. The primary goal of neural network learning is to adjust connection weights and deviations. Complete connections are frequently made in the last few layers of a CNN to allow the weighted sum of previously defined features to be calculated. Matrix multiplication, which is effectively a linear transition from one eigenspace to another, is the core operation of the full connection. In practical applications, features extracted from a feature space are usually mapped to the sample label space, and parameters are equivalent to feature weighting. However, due to parameter redundancy in the full connection layer, some models use global average pooling instead of full connections. The “flatten” layer is used to flatten the input by one-dimensionally transforming the multidimensional input, as shown in Figure 9. It is commonly employed when transitioning from the convolutional layer to the fully connected layer.

However, in transfer learning, the full connection layer can guarantee the transfer of the model representation capability. The influence parameters of the full connective layer on the model include the total number of the full connective layer, the number of neurons of a single full connective layer, and the activation function.

### 4.3. Activation Function and Loss Function of Convolutional Network

Adding an activation function to a neural network can increase the model’s nonlinear expression ability. Making data linearly separable can be solved by introducing nonlinear functions and linear transformations. To prevent a simple linear combination, an activation function such as sigmoid, tanh, ReLU, or others is introduced after each layer’s output to transform the current feature space into another space via linear mapping, allowing the data to be better categorized.

#### 4.3.1. Sigmoid Function

**Figure 10:**
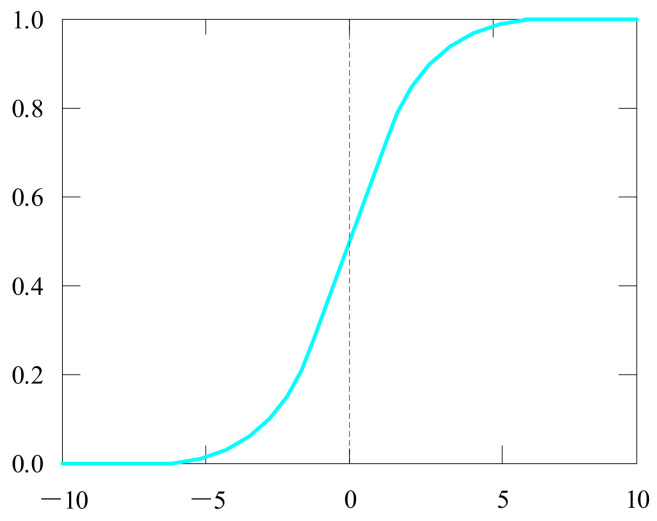
Sigmoid curve image.

The sigmoid activation function [45,46] has values between 0 and 1, an S-shaped curve as shown in Figure 10.

(16) $$f\left(x\right)=\frac{1}{1+e^{-x}}$$

It is simple to comprehend and use. However, it has certain flaws, such as gradient vanishing, and its output is not centered on zero. Therefore, its gradient updates in different directions and goes too far. The sigmoid functions tend to saturate quickly and make the gradient disappear.

#### 4.3.2. Tanh Function

**Figure 11:**
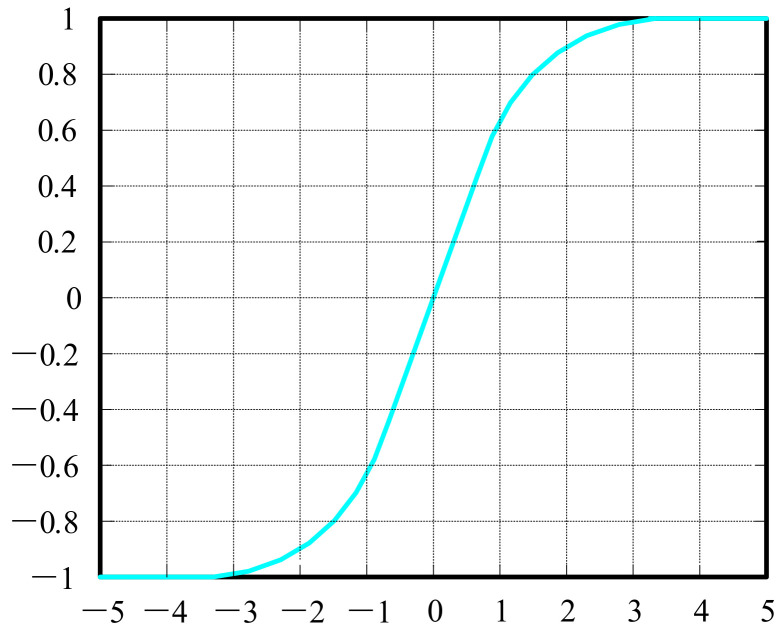
The tanh curve image.

To overcome the sigmoid function’s problem, the hyperbolic tangent function [47,48] shown in Figure 11 was introduced to solve the problem of the sigmoid function. Its output is zero-centered because its values range from −1 to 1. However, −1 makes the tanh face the same problem of slow convergence.

(17) $$f\left(x\right)=\tan hx=\frac{\sin hx}{\cos hx}=\frac{e^{x}-e^{-x}}{e^{x}+e^{-x}}$$

#### 4.3.3. ReLU, Leaky ReLU, and Parametric ReLU

**Figure 12:**
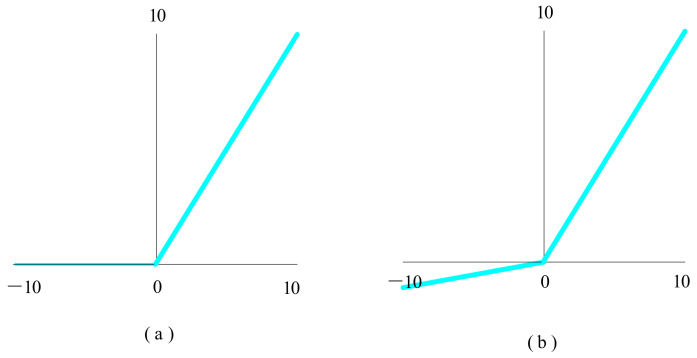
Functions of ReLU (**a**) and Leaky ReLU (**b**).

The rectified linear unit (ReLU) function [49,50], shown in Figure 12a, is used in a significant number of deep learning models to handle and fix the problem of gradient disappearance.

(18) $$\left(x\right)=\max\left(x,\;0\right)$$

where if x<0, $f\left(x\right)=0$, and if x≥0, $f\left(x\right)=x$.

However, ReLU’s constraint is that it can only be employed in the neural network model’s hidden layer. As a result, the output layer can use the softmax function to tackle the classification problem and calculate the class probability. Another issue with the ReLU function is that some gradients can be weak or even die while being trained. It has the potential to induce weight updates, making it inactive at all times.

To solve the problem that ReLU might cause neurons to die, another modification function called Leaky ReLU was introduced, which introduced a small ramp to keep the updated values alive.

(19) $$f\left(x\right)=\left\{\begin{array}{cc} 0.01x & \mathrm{for}\;x<0\\ x & \mathrm{for}\;x\geq 0 \end{array}\right.$$

where 0.01 is the slopes.

The Leaky ReLU nonlinear function [51] is shown in Figure 12b. The purpose of this is not to lose all the negative axis information, and to solve the problem of “death” of ReLU neurons.

A further approach is Parametric ReLU (PReLU) [52], in which a parameter ∅ is created that changes based on the model for x<0. The formula is as follows:

(20) $$f\left(x\right)=\left\{\begin{array}{cc} x & \mathrm{if}\;x>0\\ \emptyset x & \mathrm{otherwise} \end{array}\right.$$

**Figure 13:**
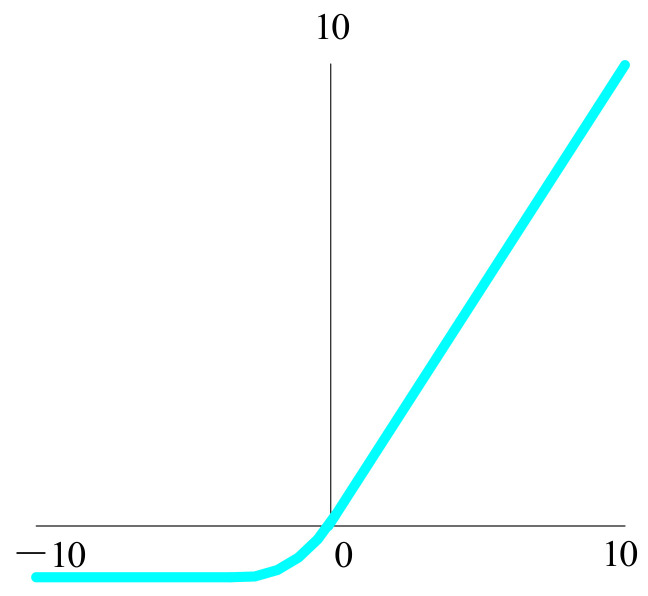
The function of the exponential linear unit.

The exponential linear unit [53] was proposed to have the advantage of ReLU, as shown in Figure 13, with no dead ReLU problem and an output mean close to zero, and PReLU and Leaky ReLU share this advantage with it. The exponential linear unit is thought of as a function between ReLU and Leaky ReLU.

(21) $$f\left(x\right)=\left\{\begin{array}{cc} x & \mathrm{for}\;x>0\\ \emptyset \left(\exp\left(x\right)-1\right) & \mathrm{for}\;x\leq 0 \end{array}\right.$$

Finally, there is a ReLU version known as the MaxOut function, which is generated by combining the ReLU and the Leaky ReLU functions. Unlike the regular activation function, MaxOut can fit all possible convex functions through piecewise linear functions. MaxOut can be regarded as a learnable piecewise linear function that requires parameters because it is learnable, and the parameters can be learned through backpropagation. The operation of MaxOut is to maximize everything linearly, which means that several lines intersect as the boundary of the segment and then maximize each segment.

MaxOut is a generalization of ReLU and Leaky ReLU, and the formula for the function is as follows:

(22) $$f\left(x\right)=\max\left(w_{1}^{T}x+b_{1},\;w_{2}^{T}x+b_{2}\right)$$

MaxOut has all the advantages of linearity and the unsaturated property of ReLU, as well as strong fitting ability. It can fit any convex function. The most intuitive interpretation is that piecewise linear functions can fit any convex function with any accuracy. MaxOut, in turn, can take the maximum value of n hidden layer nodes, and these “hidden layer” nodes are linear as well. As a result, the maximum value can be regarded as piecewise linear for a wide range of values. More importantly, MaxOut does not cause neuronal necrosis. However, it is undeniable that MaxOut increases the number of parameters, resulting in too much computation. Compared with the activation function of conventional and simple functions, MaxOut also needs backpropagation to update its weight coefficient.

During model training, it is usually possible to use the ReLU function, which applies only to the hidden layer. However, the leaky ReLU or MaxOut functions can overcome this difficulty when it comes to dead neurons. Considering the serious situation of the gradient disappearance problem, the sigmoid function and tanh function are usually not applicable to the training model, and gradient disappearance will lead to more complicated problems to be solved in the training of a neural network model.

#### 4.3.4. Loss Function

When looking for the best weight and deviation parameters in neural network learning, the key goal is to identify the parameter that makes the loss function value as little as feasible. Therefore, the accuracy of model identification can be improved by adding a loss function to obtain parameters. The derivative of the parameter and the gradient are calculated, and then that derivative is used as a guide to progressively update the parameter value to identify the spot where the loss function value is as little as possible. When the loss function of a given weight parameter in a neural network is derivative, and the derivative value is negative, the value of the loss function can be lowered by moving the weight parameter to the positive direction. Conversely, if the derivative is positive, the loss function’s value can be decreased by setting the weight parameter to a negative number. However, when the derivative value is 0, no matter which direction the weight parameter changes, the value of the loss function will not change. At that time, the update of the weight parameter will stop there.

The loss function is used to determine the extent to which the model’s predicted value differs from the true value. The model’s performance improves as the loss function improves. Different loss functions are used by different models. Common loss functions include 0–1 loss function [54], absolute loss function [55,56], logarithmic loss function [57,58], quadratic loss function [59], Hinge loss function [59,60], cross-entropy loss function [61], and so on.

### 4.4. Training Methods of Convolutional Networks

Many machine learning models use gradient descent [62] or an enhanced technique based on gradient descent. The optimization problem of the model is to solve the problem of the extremum of the function, including the maximum and minimum. The instability of gradient-based learning methods in deep neural networks suggests that activation functions, weight initialization, hyperparameters, and even learning algorithm implementation all play a role. In neural network model training, gradient descent optimization is the most often used optimization approach. The gradient descent algorithm is used to train deep learning models for optimization. The backpropagation algorithm can effectively calculate gradient for the neural network model [63], which is particularly useful for gradient descent algorithm neural network training. The gradient descent algorithm’s learning rate is an important parameter [64]. A low learning rate causes the model to converge slowly, so choosing the right learning rate is crucial. On the contrary, a high learning rate may lead to training oscillations prone to large differences. For improved gradient descent, the basic conditions are exponential weighted moving average inclusion and fast convergence.

Gradient descent is divided into three categories, which differs mostly in the amount of data used. The gradient of the cost function is calculated using the entire data set in batch gradient descent. Due to the error of calculating each example of all the training data, the model of all the training samples will be updated after evaluation. The advantages of batch gradient descent are high computational efficiency, stable error gradient, and cohesion. However, error gradient stability can sometimes lead to an aggregation state defect, indicating that it is not the optimum state that the model can obtain [65]. It also necessitates having all of the training data in memory and accessible to the algorithm. The mini-batch gradient descent [66] method, which uses a batch of training samples for training each time, has become the most widely used algorithm because it can quickly obtain accurate answers [67]. This method is unique in that it calculates the gradient of the cost function using a chosen number of training samples in each iteration rather than the entire data set. This method reduces the variation of parameter updating, enables more stable convergence, and makes use of the highly optimized matrix for efficient gradient calculation. However, it may be affected by the learning rate, which easily leads to the convergence problem. Compared with batch gradient descent, stochastic gradient descent can achieve fast computation [68,69]. It randomly selects one training sample from the entire data set for each iteration in order to compute the gradient of the cost function and update the parameters. For large data sets, stochastic gradient descent also has the advantage of fast convergence [70]. However, despite the speed of the calculation results, the accuracy of the results obtained by this method may not be the best. The gradient descent algorithm can converge quickly to find the global optimum and converge to the optimal point. However, the current deep learning model is a large nonlinear structure with many local optimal points. As a result, gradient descent algorithms become less reliable.

Many researchers have proposed new methods to overcome some problems brought on by gradient descent, such as difficulty in choosing learning rate, slow computation speed, and inability to guarantee the optimal solution of convergence. Momentum optimization, proposed by Polyak [71] in 1964, is based on the assumption that when a ball rolls down a hill, its initial speed is slow, and as acceleration increases, it increases rapidly, eventually reaching a steady speed due to drag. Adagrad is a gradient descent algorithm with an adaptive learning rate proposed by Duchi et al. [72] in 2011. The learning rate is gradually attenuated over the training iteration process, with the frequently updated parameters having a faster learning rate attenuation. Hinton introduced a hyper parameter in the course to overcome the problem of fast attenuation of the learning rate based on the development of the Adagrad method. The proposed RMSProp attenuates in the accumulation of gradient square terms, which reduces the gradient explosion and thus helps to avoid the rapid decline of the learning rate. In 2014, Kingma and Ba [73] combined the Momentum and RMSProp algorithm and proposed an Adaptive Moment Estimation optimization algorithm. Compared with the former, the learning rate is self-adaptive and the impulse item is added compared with the latter. For gradient descent algorithms, the learning rate is the most important super parameter. A high learning rate may lead to non-convergence and direct differentiation of training. A small set of learning rate can achieve the purpose of convergence, but the training time may be insufficient. When a high learning rate is applied, the training speed increases dramatically as the ideal point approaches, and the oscillation becomes unstable. Therefore, the choice of different learning rates may have a significant impact on model training. In the following research, many improved algorithms also appeared [74,75,76,77].

One of the issues with training neural networks, particularly deep neural networks, is gradient disappearance or gradient explosion [78], where the gradient on the neurons becomes very small or very large, which makes training more difficult. Gradient vanishing refers to the occurrence of errors after transmission attenuation at each layer in the process of error return. The gradient on the neurons continues to decay as the number of network layers deepens, resulting in the learning speed of the hidden layer neurons being slower than that of the hidden layer neurons. The output layer, where the derivatives of the activation function are multiplied on each layer, returns the error. Where the value range of the derivative is less than 1, or even if the derivative of the sigmoid function approaches 0 in the saturated region at both ends, the gradient will continue to decay or even disappear. The opposite problem is the explosive gradient problem, in which the gradient of the anterior layer neurons becomes very large. Unlike gradient vanishing, gradient explosions are usually caused by excessive weight. In summary, gradient extinction occurs most often when a deep network is used with an incorrect loss function, whereas gradient explosion occurs when a deep network is used with an excessive weight initialization value.

### 4.5. Forward and Backward Propagation of Convolutional Networks

Each neuron in a neural network’s forward propagation [79] is separated into distinct groups based on the sequence in which it receives information, and each neuron may be thought of as a neural layer. Each layer’s neurons receive the previous layer’s output and pass it to the next layer. From input to output, all information in the network goes in one direction. A neural network is actually a function of output vector to output vector, and its forward propagation is the vector dot product, weighted sum, and through an activation function to obtain the target output value.

In forward propagation, it is assumed that a sample with a feature of $p\in R^{s}$ is input, and the deviation term is not considered, so the intermediate variable is:

(23) $$v=W^{\left(1\right)}p$$

where $W^{\left(1\right)}\in R^{s\times l}$ is the hidden layer’s weight parameter. The hidden layer variable with vector length l is obtained after the intermediate variable $v\in R^{l}$ is input into the activation function ∅:

(24) $$l=\emptyset \left(v\right)$$

Assuming that the output parameter only has the weight $W^{\left(2\right)}\in R^{h\times l}$, the output layer variable of vector length h can be obtained:

(25) $$o=W^{\left(2\right)}l$$

Assuming ℓ is the loss function and the sample label is q, the loss of a single data sample can be calculated:

(26) $$L=\ell \left(o,q\right)$$

The regularization term for the hyperparameter λ is:

(27) $$r=\frac{\lambda }{2}\left(||w^{\left(1\right)}{||}_{F}^{2}+\;||w^{\left(2\right)}{||}_{F}^{2}\right)$$

The Frobenius norm of the matrix is represented by F. The loss of the final model with regularization on a given data sample is:

(28) K=L+r

**Figure 14:**
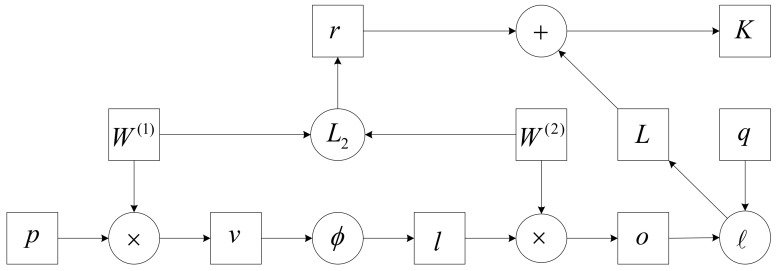
Computational diagram of forward propagation.

K is the objective function of a given sample of data. The calculation of forwarding propagation is shown in Figure 14.

Backpropagation [80] is a monitoring algorithm that tags data. Neurons can receive not just the signals of other neurons but also their own feedback signals as neural networks propagate. This method allows cost function information to flow back through the network, recalculate the gradient of the loss function for network ownership, and fee back the optimization procedure, changing the weights to minimize the loss function. The loss function is used before backpropagation to quantify the difference between the output calculated by the training sample and the actual output of the training sample. Therefore, the backpropagation is actually the understanding of the process of weight and deviation changes affecting the loss function, that is, the calculation of partial derivatives.

Supposing that for input or output, X, Y, and Z are functions of tensors of any shape, $Y=f\left(X\right)$, and $Z=g\left(Y\right)$, by the chain rule, we have:

(29) $$\frac{\partial Z}{\partial X}=\mathrm{prod}\left(\frac{\partial Z}{\partial Y},\frac{\partial Y}{\partial X}\right)$$

The prod operator multiplies two inputs based on their shape, transpose, and switch input positions. We have two parameters, $W^{\left(1\right)}$ and $W^{\left(2\right)}$, and the backpropagation target calculations are $\partial K/\partial W^{\left(1\right)}$ and $\partial K/\partial W^{\left(2\right)}$, respectively. Calculate the gradient of the relative loss terms L and r of the objective function K=L+r.

(30) $$\frac{\partial K}{\partial L}=1,\;\frac{\partial K}{\partial r}=1$$

Using the chain rule, calculate the objective function’s gradient $\partial K/\partial o\in R^{h}$ with regard to the output layer variable:

(31) $$\frac{\partial K}{\partial o}=\mathrm{prod}\left(\frac{\partial K}{\partial L},\frac{\partial L}{\partial o}\right)=\frac{\partial L}{\partial o}$$

Then, calculate the gradient of two parameters related to the regular term:

(32) $$\frac{\partial r}{\partial W^{\left(1\right)}}=\lambda W^{\left(1\right)},\frac{\partial r}{\partial W^{\left(2\right)}}=\lambda W^{\left(2\right)}$$

The gradient $\partial K/\partial W^{\left(2\right)}\in R^{h\times l}$ that is closest to the output layer’s model parameters can then be calculated:

(33) $$\frac{\partial K}{\partial W^{\left(2\right)}}=\mathrm{prod}\left(\frac{\partial K}{\partial o},\frac{\partial o}{\partial W^{\left(2\right)}}\right)+\mathrm{prod}\left(\frac{\partial K}{\partial r},\frac{\partial r}{\partial W^{\left(2\right)}}\right)$$

Backpropagation continues along with the output layer to the hidden layer, and the gradient of the hidden layer variable $\partial K/\partial l\in R^{l}$ is:

(34) $$\frac{\partial K}{\partial l}=\mathrm{prod}\left(\frac{\partial K}{\partial o},\frac{\partial o}{\partial l}\right)$$

The gradient $\partial K/\partial v\in R^{l}$ of the intermediate variable v is:

(35) $$\frac{\partial K}{\partial v}=\mathrm{prod}\left(\frac{\partial K}{\partial l},\frac{\partial l}{\partial v}\right)=\frac{\partial K}{\partial l}⊙\emptyset^{\prime }\left(v\right)$$

where ⊙ is the element multiplication operator. Finally, the gradient of the model parameters closest to the input layer is obtained:

(36) $$\frac{\partial K}{\partial W^{\left(1\right)}}=\mathrm{prod}\left(\frac{\partial K}{\partial v},\frac{\partial v}{\partial W^{\left(1\right)}}\right)+\mathrm{prod}\left(\frac{\partial K}{\partial r},\frac{\partial r}{\partial W^{\left(1\right)}}\right)$$

The proposal of backpropagation speeds up the calculation speed of the model, but it shows a shortcoming in the application of multi-hidden layer networks because it is a multi-layer network for nonlinear differentiable function weight training. The algorithm can make the network weights converge to a solution, but it cannot guarantee that the obtained solution is the error hyperplane, and the high-probability solution is the local minimum solution.

### 4.6. Network Architecture

#### 4.6.1. AlexNet

Popular in computer vision for convolutional neural networks is AlexNet, developed by Krizhevsky et al. [36]. AlexNet outperformed the runner-up in the 2012 ImageNet ILSVRC Challenge, with an accuracy rate of 57.1% and a Top-5 recognition rate of 80.2%. This CNN is comparable to LeNet in terms of design, but it is deeper, larger, and contains convolutional layers stacked on top of each other. AlexNet has three fully linked layers and five convolutional layers. A convolutional layer is frequently followed by a pooling layer in the prior CNN construction. ImageNet, a dataset with over 15 million tagged high-resolution photos in around 22,000 categories, was employed in the experiment. The activation function of neurons is generally sigmoid or tanh, whereas in AlexNet, ReLU is selected, and the training speed of this activation function is several times faster than that of the traditional neural network.

(37) $$\mathrm{ReLU}\left(x\right)=\max\left(x,\;0\right)$$

In the experiment, a four-layer CNN was used to test the CIFAR-10 data set. When the error rate of the training set reached 25%, the speed of using ReLU was 6 times faster than that of tanh.

AlexNet uses local response normalization (LRN) in addition to the activation function, which simulates a neurobiological function called lateral inhibition by creating competition mechanisms for local neuronal activity, making the larger values of response larger than the larger ones. It also inhibits neurons with smaller feedback, which improves the model’s generalization ability. LRN, in addition to the activation of ReLU, increases the network’s generalization capability and reduces the error rate by 1% on ILSVRC-2012. LRN only normalizes the adjacent regions of the data without changing the size and dimension of the data. The formula is as follows:

(38) $$b_{x,y}^{i}=a_{x,y}^{i}/{\left(k+\alpha \overset{\min\left(N-1,i+\frac{n}{2}\right)}{\underset{j=\max\left(0,\;i-\frac{n}{2}\right)}{\sum }}{\left(a_{x,y}^{j}\right)}^{2}\right)}^{\beta }$$

where $a_{x,y}^{i}$ represents the value generated by the ith convolution at (*x*,*y*), and then the result of the ReLU activation function is applied; n denotes the number of neighboring convolution cores, and N is the total number of convolution cores at this layer. k, n, α, and β are all hyperparameters, and their values are obtained from the verification set experiments: k=2, n=5, α=0.0001, β=0.75.

The normalization process uses lateral inhibition to provide a competitive mechanism for local neural activity, enhancing the model’s generalization ability by making the response to bigger values larger and decreasing the response to smaller values. It can make the features more obvious, the implementation more obvious, and contribute to the efficient recognition of the model. However, it has been proven that the effect of local normalization is not obvious, and it is almost ignored in subsequent experiments.

Maximum overlapping pooling is used in CNN. Prior to that, CNN utilized average pooling, and AlexNet used maximum pooling, but overlapping pooling prevented the blurring effect of average pooling. Furthermore, AlexNet proposes that the stride be lower than the size of the pooled core, resulting in overlap and coverage between the pooled layer’s outputs, which improves the richness of the features to some extent. The pooling layer is the output of the surrounding neurons in the same convolutional nucleus domain. A grid of pool cells with a spatial distance of s pixels is designated as the pooling layer. It is also known as dividing the previous convolution layer’s results into blocks of the same size as the step size and summing the convolution mapping results with the block size. When summing up the extracted characteristics of the overlapping pooling, the input of the nearby pooling units will be influenced. The extracted results from max-pooling may be duplicated. Furthermore, the experiment revealed that overlapping pooling has a better effect than traditional pooling, increasing by 0.4% in Top-1 and 0.3% in Top-5, respectively, avoiding overfitting in the training stage.

In AlexNet’s data enhancement, random crop and flip were used to expand the number of training samples by thousands of times, which also made random cropping a universal method. Another enhancement was the strength of the conversion RGB channels, which was rarely used in later models, and for this model, the error rate was reduced by about 1%.

The full-connection layer, which has too many parameters and is prone to overfitting, bears the brunt of the CNN’s computational load. Dropout makes some nodes fail randomly during training and reduces the risk of overfitting by not contributing connection weight and forcing these neurons to learn complementary features. Dropout is achieved in a neural network by changing the structure of the neural network. By specifying the probability for a neuron at a specific layer, the neuron is set to 0 and does not participate in either forward or backward propagation. It is as if a node in the network is removed, but the number of neurons in the input and output layers remains unchanged. The parameters are then adjusted based on the neural network’s learning mechanism. In the next cycle, some neurons are deleted at random until the training is completed. Dropout can also be thought of as a mix of models, with different network architectures being formed at different times. Overfitting can be substantially reduced by mixing numerous models. Dropout requires just twice the amount of training time to accomplish the effect of model combination and increases the speed of operation. All of the AlexNet layer parameters are shown in Table 2.

Compared with the neural network before AlexNet, it improves data enlargement, adds dropout to prevent overfitting problems effectively, and uses ReLU to replace the traditional sigmoid and tanh activation functions to reduce the complexity. Local response normalization simulates the lateral inhibition function of biological neural networks, improves the model generalization ability, and reduces the overfitting of the system. It also divides the network into upper and lower parts by using multi-GPU parallel training to improve the training speed.

#### 4.6.2. VGGNet

**Figure 15:**
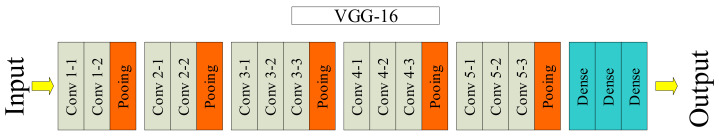
The structure of the VGG-16.

The basic network in the 2014 ImageNet competition, VGGNet [81], proposed by the Visual Geometry Group at the University of Oxford, won first place for the location job and second place for the classification objective. This method is offered to demonstrate that raising the network’s depth has some impact on the network’s final performance. The larger convolution cores 11 x 11, 7 x 7, and 5 x 5 of AlexNet were replaced by numerous consecutive 3 x 3 convolution cores, which is an improvement over AlexNet. The major goal is to increase the depth of the network and the effectiveness of the neural network to a certain amount while maintaining the same perceptual area. Because multiple nonlinear layers can increase the depth of the network and ensure the learning of more complex patterns for a given receptive field, using stacked small convolution kernels is better than using large convolution kernels for a given receptive field, and the cost is relatively low with fewer parameters. Figure 15 shows the network structure of VGG-16.

VGG has the advantage of having a fundamental structure, as the entire network utilizes the same convolution kernel size and maximum pooling size. A group of small filter convolutional layers is preferable to a single large filter convolutional layer. VGG’s downside is that it consumes more computer resources and employs more parameters, resulting in increased memory use.

#### 4.6.3. GoogLeNet

GoogLeNet [82] introduced Inception, a structure that grouped sparse matrices into relatively dense submatrices to boost computing speed while maintaining the sparsity of the network topology. The fundamental idea is to approximate the best local sparse structure using dense components. Increasing network depth and width, which involves a large number of parameters, is the most basic technique to improve network performance. The huge number of parameters, on the other hand, makes overfitting easy and significantly increases the amount of calculation required. The transformation of complete connection and even general convolution into sparse connection is the primary approach for solving the problem of model overfitting and massive computation. Traditional networks all employ random sparse connections to disrupt network symmetry and increase learning ability. However, because calculating non-uniform sparse data is wasteful on computer hardware and software, the complete connection layer has been re-enabled since AlexNet to better optimize parallel operations. The network structure of GoogLeNet is shown in Table 3. The number of 1x1 filters utilized after pooling in an inception module is shown in the “PP” column [82].

In GoogLeNet, using different-sized convolution kernels means using different-sized receptive fields. Finally, the stitching combines the characteristics of several scales. The convolution kernel sizes are 1, 3, and 5, mostly to aid feature stitching and alignment. After convolution, if the padding = 0, 1, and 2, convolution = 1 is set, the features of the same dimension may be produced, and then those characteristics can be immediately spliced together. The characteristics get more abstract as the network is studied in more depth, and the receptive area involved in each feature grows bigger. Consequently, as the number of layers grows, the fraction of convolution grows as well, resulting in a massive amount of computation.

The stated goal of GoogLeNet is to expand the network while maximizing computing power. GoogLeNet does a good job of handling image categorization, but it immediately increases computational overhead when trying to build a larger network by simply scaling up the Inception structure. GoogLeNet Inception v2 [83] is proposed to address the expression bottleneck at the front of the network. Information flow is unable to pass through highly compressed layers as it propagates forward. At the same time, the more distinguishable problem with high dimensional features is considered to accelerate the training, and the spatial aggregation is carried out on the low-dimensional embedding without worrying about losing a lot of information. The information can be simply compressed. Then the training will be accelerated.

The Inception v2 network is a step forward from the original Inception network. On the one hand, learning VGG substitutes two 3 x 3 convolutions for the 5 x 5 convolution in the Inception module, reducing the number of parameters and speeding up the calculation. The article, on the other hand, suggests a batch normalization (BN) layer. When BN is employed in a layer of a neural network, it reduces the internal variate shift by processing the normalization of the output to the normal distribution of $N\left(0,1\right)$ by normalizing the internal process of each mini-batch data. BN also acts as a regularization, so the dropout can be reduced or eliminated to simplify the network structure. Some corresponding adjustments can make the gain of BN more obvious: (i) increasing the learning rate and speeding up the learning decay rate to apply to the normalized data of BN, (ii) removing dropout and reducing L2 regularity, and (iii) removing LRN, plus more thorough shuffling of the training samples and reducing optical distortion of the data during data enhancement. Inception v2 is 14 times quicker in training to the accuracy of Inception v1, and the model has a greater upper limit of accuracy when convergent after applying these metrics.

Factorization, which decomposes a big two-dimensional convolution into two smaller one-dimensional convolutions so that the surplus computational power may be utilized to deepen the network and speed up calculations, is one of the most fundamental advances in Inception v3 [84]. Furthermore, the convolution layer is divided to improve the network’s depth, increase its nonlinearity, and deal with more and richer spatial data to increase the variety of features.

#### 4.6.4. ResNet

In 2015, residual network (ResNet) was proposed by He et al. [85] from Microsoft Research Asia, which solved the difficulty of deep network training and won five first prizes in the ILSVRC and COCO 2015 competitions. The network’s depth is critical to the model’s success. When the number of network layers is raised, the network may extract more complicated feature patterns. Therefore, theoretically, when the model is deeper, better results can be produced. The experiment, however, showed that the deep network is vulnerable to deterioration. Despite the employment of various technical measures to relieve the gradient disappearance or explosion difficulties that exist in the deep network, the deep network degradation problem remains tough to fix. The accuracy of the network becomes saturated or even declines as the depth of the network rises.

**Figure 16:**
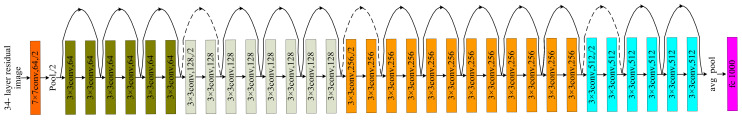
The structure of ResNet.

The proposal of the residual network is based on solving the degradation problem. Its structure is shown in Figure 16. For an accumulation layer structure, when the output is x, its learned feature is marked as $H\left(x\right)$, and the residual formula is:

(39) $$F\left(x\right)=H\left(x\right)-x$$

The original learning feature is $F\left(x\right)+x$. Direct learning using original features is more difficult than residual learning. When the residual is 0, the identity mapping is done by the accumulation layer, and the network performance is not affected. Meanwhile, to improve speed, the accumulation layer learns new features depending on the input characteristics. The residual element can be written as follows:

(40) $$y_{n}=h\left(x_{n}\right)+F\left(x_{n},W_{n}\right)$$

(41) $$x_{n+1}=f\left(y_{n}\right)$$

where $x_{n}$ and $x_{n+1}$ represent the input and output of the n-th residual element, respectively. The learned residuals are represented by F, whereas the identity mapping is represented by $h\left(x_{n}\right)=x_{n}$. The ReLU activation function is denoted by f. Then we calculate the learning characteristics of n from shallow layer to deep layer N as follows:

(42) $$x_{N}=x_{n}+\sum_{i=n}^{N-1}F(x_{i},W_{i})$$

To find the gradient of the reverse process, use the chain rule:

(43) $$\frac{\partial loss}{\partial {𝓍}_{n}}=\frac{\partial loss}{\partial {𝓍}_{N}}\cdot \frac{\partial {𝓍}_{N}}{\partial {𝓍}_{n}}=\frac{\partial loss}{\partial {𝓍}_{N}}\cdot \left(1+\frac{\partial }{\partial {𝓍}_{N}}\sum_{i=n}^{N-1}F({𝓍}_{i},W_{i})\right)$$

#### 4.6.5. EfficientNet

EfficientNet [86] is scalable and efficient with its network depth, network width, and image resolution. Unlike other network architectures, the usual network architecture is designed to increase accuracy by amplifying either dimension. EfficientNet is one of the most reliable, accurate, and best performing networks in the market by finding the best connections in all three dimensions. The amplification of any of the three dimensions may lead to an improvement in accuracy, but as the multiplier of the experiment increases, the improvement in accuracy decreases. Among the three aspects of expansion dimension, as for the expansion of depth, network depth is used in many convolutional networks to capture richer and more complex features, but there may be a problem of gradient disappearance. Several approaches have been attempted to address the training issue, but the deep network’s accuracy suffered as a result. The expansion of network width can capture more fine-grained features, which are easy to train. However, the shallow wide network is prone to difficulties in capturing high-level features, leading to premature accuracy saturation and precision reduction. High-resolution input images can make the convolutional neural network receive finer image details, but too-high resolution may also lead to lower network accuracy.

The whole convolution network of EfficientNet is N, and the function mapping of the *j*-th convolution layer is:

(44) $$Y_{j}=f_{j}\left(X_{j}\right)$$

$Y_{j}$ is the output tensor, $X_{j}$ is the input, and the dimension is $\left\{H_{j},\;W_{j},\;C_{j}\right\}$. The whole convolution network consisting of l convolution layers N is:

(45) $$N=f_{l}⊙\ldots ⊙f_{2}⊙f_{1}\left(X_{1}\right)={⊙}_{i=1\ldots l}f_{i}\left(X_{1}\right)$$

Using a stage to represent units of multiple convolution layers with the same structure, the convolution network N is expressed as:

(46) $$N=\underset{j=1\ldots .s}{⊙}f_{j}^{L_{j}}\left(X_{\left\{H_{j},\;W_{j},\;C_{j}\right\}}\right)$$

$\left\{j=1\ldots .s\right\}$ represents the sequence number of the stage and $f_{j}^{L_{j}}$ represents the j-th stage, which is composed of the convolution layer $f_{i}$ repeated $L_{j}$ times.

The authors fixed the basic structure of the network to reduce the search space, only changing the network depth, width, and input resolution. In the limitation, the network amplification can only be multiplied by the optimized constant multiplier based on the primary network. Assuming that w is the ratio of network width, h is the ratio of network depth, and r is the ratio of the resolution, then the abstract mathematical model is:

(47) $$\underset{d,\;h,\;r}{\max}ACC\left(N\left(d,h,r\right)\right)$$

(48) $$s.t.\;N\left(d,h,r\right)=\underset{j=1\ldots .s}{⊙}{\hat{f}}_{j}^{d\cdot {\hat{L}}_{j}}\left(X_{\left(r\cdot {\hat{H}}_{j},\;r\cdot {\hat{W}}_{j},\;w\cdot {\hat{C}}_{j}\right)}\right)$$

(49) $$Memory\left(N\right)\leq target\_memory$$

(50) $$FLOPS\left(N\right)\leq target\_flops$$

During the experiment, the authors put forward a compound scaling method that balances the network width and depth with the image resolution. With speed and accuracy, EfficientNet stands out as a practical model compared to other network architectures.

### 4.7. Advantages of Tumor Diagnosis Based on Convolutional Neural Network

The most obvious difference between the convolutional neural network and the traditional neural network is that feature extraction is more inclined to automatic extraction by machine than by manual means. Although manual feature extraction can be simple and effective for specific simple tasks, it cannot be used as a general approach. The biggest advantage of CNN is feature extraction. Because CNN’s feature detection layer learns from training data, it eliminates display feature extraction and instead learns implicitly from the training data. Furthermore, because the weight of neurons on the same feature mapping surface is the same, the network may learn in tandem, which is another significant benefit of a convolutional network over a fully linked network. Local perception and parameter sharing are the essential features of CNNs, which are mostly employed to detect displacement, scaling, and other types of distortion. The organization of the visual system in biology generated the notion of the local perceptual field. Local information is received by neurons in the visual cortex. They just need to perceive local information and then integrate it at a higher level in order to obtain global knowledge. The feature extraction technique is analogous to the bottom-up method, in which local input is received layer by layer and then aggregated constantly. Because of its unique structure of local sharing, the convolutional weighted neural network offers distinct benefits in voice recognition and picture processing. Its design is more akin to a biological neural network, and the sharing of weights decreases the network’s complexity. In particular, multidimensional network input vector pictures may directly input the feature, obviating the need for data reconstruction in feature extraction and classification.

The traditional neural network is replaced by the intelligent automatic processing ability of CNN by the need for human intervention. According to many experimental results, the CNN has good experimental performance and strong learning ability. Because the CNN has enough layers, the model covers a wide range and can be mapped to any function theoretically to solve more complex problems. A CNN is heavily reliant on data, and the more data there is the greater the model’s performance. The task of tumor image recognition even has a tendency to exceed human performance, and the upper limit of the ability of the model to recognize images can be improved by adjusting parameters. However, a CNN requires a lot of data calculation, which leads to high cost, cannot be solved on mobile devices, and has a high CPU demand. Moreover, the model design of a CNN is more complex than the model design of a traditional neural network, and the over-reliance on data leads to low interpretability. In the case of unbalanced training data, problems such as discrimination and neglect of results may occur.

## 5. Practical Applications of Convolutional Neural Network in Tumor Diagnosis

A systematic literature search was conducted in the following electronic databases: PubMed via Medline, AMED, EMBASE, CINAHL. The search words are “Convolutional Neural Networks”, “lung tumor”, “brain tumor”, “tumor detection”, “the application of tumor detection”. All databases have no language or functional limitations.

### 5.1. Lung Tumor

The main causes of lung tumor are smoking, occupational and environmental contact, ionizing radiation, chronic lung infection, air pollution, and genetic and other factors. Terminal non-small-cell lung cancer patients have fatigue, weight loss, decreased appetite, and other manifestations, and dyspnea, cough, hemoptysis, and other local symptoms appear [87]. The nerves in the bronchi are pretty sensitive, and cancer irritates the bronchi, causing the patient to cough, and over time, the symptoms of hemoptysis will likely develop. If lung tumors make up the majority of lung cancer, it is a severe threat to the patient’s breathing. Lung tumors that develop into lung cancer can also make it so the patient’s sputum cannot be removed, leading to bacterial pneumonia and fever, which is called obstructive pneumonia. At the same time, the patient’s trachea is blocked; the normal alveolar sac cavity disappears, affecting the exchange of oxygen and carbon dioxide; and the patient will have a feeling of chest tightness and shortness of breath. Chest pain is one of the more common risks of lung cancer, and pleural effusion may also occur. Pleural fluid puts more pressure on the lungs, increasing the patient’s breathing difficulty, and is difficult to treat.

Deeplung is an automatic lung computed tomography cancer diagnostic method proposed by Zhu et al. [88] Deeplung is divide into two parts: nodule identification and classification. Nodule detection determines the location of candidate nodules, and classification determines whether the nodules are benign or cancerous. Two deep 3D DPNs were built for nodular identification and classification, taking into account the 3D character of lung CT data and the compactness of dual-path networks (DPNs). 3D faster regions with CNN (R-CNN) are explicitly created for nodule detection with 3D dual-path blocks and encoder–decoder structures like U-NET to learn nodule features effectively. A gradient boosting machine (GBM) with 3D dual-path network characteristics is presented for nodule classification. The LIDC-IDRI public dataset is used to validate the nodule classification network, and its classification performance is superior to current approaches and exceeds experienced clinicians using imaging modalities. Candidate nodules are identified initially in the Deeplung system via the nodule detection subnet, and then nodule diagnosis is performed via the categorized subnet. A large number of experimental results showed that Deeplung performed as well as experienced physicians in both nodular and patient-level diagnoses on the LIDC-IDRI dataset.

Vijh et al. [89] suggested a hybrid bionic approach that combines the benefits of whale optimization and adaptive particle swarm optimization. In this method, 120 lung CT images were preprocessed and segmented using CNN to obtain segmented nodules in tumor and non-tumor areas. Whale optimization techniques and adaptive particle swarm optimization techniques were used to optimize the features. Embedded linear discriminant analysis grouped features to reduce the dimension of subsets. Various classification algorithms, such as support vector machine, artificial neural network, and CNN, were compared during the experiment, and the accuracy, sensitivity, and specificity performance scores were 97.18%, 97%, and 98.66%, respectively.

Lu [90] developed a deep CNN-based computer-aided diagnosis technique for lung tumors. The intricate and hazy CT scans of the lung were used to create this deep CNN. Simultaneously, the author discussed the relationship between model parameters and the recognition rate. He also explored the impact of different model architectures on lung tumor recognition and the different pooling method optimization strategies on deep CNN performance. Finally, the experimental findings showed that the gradient descent approach with elastic momentum had 96.4% accuracy, 97.6% sensitivity, and 95.20% specificity, proving the practicality of a deep CNN for computer-aided diagnosis of lung cancers.

Rani and Jawhar [91] proposed a method of enhanced deep CNN. The method uses a deep CNN to distinguish tumor images from the LIDC database and internal clinical images by measuring tumor image regions based on Advance Target Map Superpixel’s region segmentation and nanoscale imaging theory. Compared to previous classification algorithms tested at various stages of lung tumor pictures, the accuracy, sensitivity, and specificity scores were 97.3%, 94.9%, and 100%, respectively.

Teramoto et al. [92] developed an automated method to classify lung cytological images. Firstly, the images of lung tumor cytological specimens were enhanced with data. Based on the fine-tuning VGG-16 model, the cytological images were classified into three categories using the DCNN architecture. The accuracy, sensitivity, and specificity of classification performance were 87.0%, 83.3%, and 89.3%, respectively, via the triple cross-validation method.

Shi et al. [93] used CT and PET pictures of lung tumors as test subjects for a CNN-based multimodal recognition system for lung imaging. In lung tumor recognition, the integrated CNN can enhance recognition accuracy while reducing training time. The CNN is effective at identifying lung tumor images, according to experimental results. The number of iterations and batch size affect lung tumor recognition throughout the training process. However, the built integrated CNN outperformed the simple CNN in terms of accuracy and time consumption.

Hossain et al. [94] offered an automated workflow for detecting and segmenting lung tumors from NSCLC radiometric data sets using 3D lung CT scans. For tumor segmentation, he proposed an expanded hybrid 3D CNN architecture. Binary classifiers were first applied to select CT slices containing tumor portions and transfer the slices to segmentation models. After a 3D convolution stack, the extended convolution model pulled feature images from each 2D slice and merged CT scan information into output. Finally, the segmentation mask was cleaned by a post-processing block of morphological operations. The suggested segmentation model outperformed the recent U-Net and LungNet in terms of dice coefficient scores.

Wang et al. [95] used CNN to provide accurate quantitative analysis and computer-aided diagnosis of PET/CT pictures of lung tumors. The parameter transfer method was used to construct three CNN recognitions of lung tumor-based CT, PET, and CT/PET pictures. Then, based on the impact of several critical parameters on the recognition rate and training time, the model parameters suitable for CNN training were obtained. Finally, the three CNN integrals constructed were constructed to identify the PET/CT of lung tumor by the relative majority voting method. The performance of an integrated CNN is superior to that of a single CNN, according to experimental studies.

Tahmasebi et al. [96] proposed a CNN that automatically tracks tumor boundaries during radiotherapy to solve the difficulty of depicting lung tumors from adjacent tissues in MRI. The modified Dice metric is employed as a cost function in the CNN architecture to achieve accurate tumor region segmentation. Over 600 photos were analyzed, and the suggested technique outperformed previous sophisticated methods in terms of accuracy in showing moving tumors, with an average Dice score of 0.91 ± 0.03 compared to expert manual profiles.

Lin et al. [97] introduced GAN to enhance lung CT pictures and alleviated the problem of sparse medical pictures. With AlexNet as the trunk classification network, Taguchi’s parameter optimizer was used to find the best combination of network parameters for lung tumor classification. When compared to other state-of-the-art CNN, the optimal parameter combination’s accuracy reached 99.86%. The overall accuracy of CNN was increased by 2.73% through GAN data enhancement.

Nair et al. [98] believed that MRI is difficult in depicting and identifying lung tumors in adjacent tissues because the area of interest is similar to the surrounding area. He proposed an automatic lung tumor recognition technique based on CNN and the improved Dice metric to avoid patient deviation during radiotherapy. The experiment used 600 images similar to medical experts to obtain recognition scores with 90–95% accuracy.

Teramoto et al. [99] presented a new approach for reducing false positives and utilized CNN to detect lung nodules in PET/CT data. First, a contrast enhancement filter with a deformable kernel shape was used to detect large regions of CT and PET images of pulmonary nodules, which was coupled with high uptake areas found in PET and CT images. An integrated method was utilized to remove false positive candidates. Shape/metabolic feature analysis and CNN were used to extract features used in a two-step classifier based on rules and support vector machines. In an experiment, the authors assessed detection performance using 104 PET/CT images of lung nodules obtained from a cancer screening program, obtaining a sensitivity of 97.2% for initial detection candidates. The proposed approach for reducing false positives resulted in a sensitivity score of 90.1%, removing about half of the false positives seen in prior research.

Gan et al. [100] used the hybrid CNN technology of 2D CNN and 3D CNN to autonomously outline CT images of malignant lung tumors. The V-NET model extracts tumor context information from CT sequence pictures. 2D CNN is based on the dense connection technique, which is the encoder–decoder structure that increases information flow and enhances feature propagation. The hybrid module combines 2D and 3D features. The author compared the merged CNN with two independent CNNs. On a data set of 260 instances, training and testing could reach a median of 0.73, and the Dice measure’s mean and standard deviation were 0.72 ± 0.10. When comparing assessment indicators, it was clear that the hybrid network outperformed single 3D CNN and 2D CNN segmentation performance, which has great application potential. A deep CNN with transfer learning was utilized at various picture sizes to investigate benign nodules, primary lung cancer, and metastatic lung cancer.

Nishio et al. [101] devised a computer-aided diagnostic (CADx) approach to categorize benign nodules, primary lung cancer, and metastatic lung cancer. They found that a deep convolutional neural network had more advantages in CADx triadic classification than the traditional method of manual feature extraction and machine learning. The database of 1240 patients comprised CT scans and clinical data from 1236 individuals. Support vector machines were used in the classic CADx approach to achieve classification goals. VGG-16 with and without transfer learning (TL) was used to test the proposed DCNN approach. The DCNN hyperparameters were optimized using the random search approach in this case. The comparison of the best average validation accuracy between the traditional method and the DCNN method with or without transfer learning was 55.9%, 68.0%, and 62.4%, respectively. This approach demonstrated that DCNN is considerably superior to standard methods and that DCNN utilizing transfer learning improved accuracy. Experiments also demonstrated that using a high picture size as input information improved the classification accuracy of lung nodules.

Moitra and Mandal [102] proposed AJCC (The American Joint Committee on Cancer) classification of non-small-cell lung cancer based on the combination of fast recurrent neural network (RNN) and CNN in order to assist treatment plan and prognosis of patients. They thought deep networks could outperform standard artificial neural networks in terms of accuracy. Firstly, the image was preprocessed by resizing and enhancing, and the image texture was segmented by using maximally stable extremal regions and the speeded-up robust features. The CNN-RNN model was combined with AJCC staging information to classify the lung image.

In summary, authors who have used CNNs in lung tumor areas in recent years, and their proposed models and experimental results are shown in Table 4.

### 5.2. Brain Tumor

The brain is full of nerve tissue, and when a brain tumor develops, it tends to severely limit the space in the brain tissue. Brain tumor growth in any part of the skull can compress the intracranial nerve and then produce pain and pathological changes in the function of the affected nerve site, such as aphasia, blindness, imbalance, and so on [103]. Meningioma, which mainly grows in the skull [104], is a benign tumor that grows out on the meninges. In general, this benign tumor can cause enormous harm, and different symptoms can be developed that depend on the growth location of the tumor. [105]. Most human sensory nerves and other functional nerves need to pass information through the brain, but if they are affected by meningioma, compression of the brain nerves or tissues will easily lead to the dysfunction of related nerves. Some patients may lose their sense of hearing and smell, whereas others may have severe motor dysfunction.

Toğaçar et al. [106] established the CNN model of BrainMRNet on the basis of attention module and super column technology. The image enhancement technology-based attention module may identify the essential regions of a picture and send it to the convolution layer. The characteristics gathered from each layer of the model are maintained by the array structure of the last layer thanks to super column technology. The BrainMRNet model was tested using publicly available MRI scans of brain malignancies. Simultaneously, the accuracy of similar models such as AlexNet, GoogleNet, and VGG-16 were compared with this model, and the accuracy of this model was 96.05% greater than the first three.

Sajjad et al. [107] proposed a CNN-based multi-level brain tumor categorization method. When MRI was processing with multi-level brain tumor classification, deep learning approaches would separate tumor areas in MR images and apply data augmentation training models to decrease the shortage of data. The pre-trained CNN model for brain tumor classification was fine-tuned using enhanced data. The suggested system achieved accuracies 87.38% and 90.67%, respectively, on both the original and improved data.

Saxena et al. [108] proposed a CNN-based transfer learning (TL) method. After data expansion, the ResNet-50 model, VGG-16 model, and Inception v3 model were used to scan and categorize 253 MRI images of brain tumors. A 90% accuracy of the VGG-16 model Inception v3 model 55% and a 95% accuracy of the RESNET-50 model were obtained in the experiment.

Pashaei et al. [109] utilized a CNN and an extreme learning machine method to extract hidden characteristics from brain tumor pictures. The pictures were subsequently classified using the retrieved features by the kernel extreme learning machine (KE). The experiment used three types of brain tumor images, and the results of CNN and kernel limit learning integration were compared to other classifiers such as support vector machine, radial basis function, and others, with the accuracy rate of 93.68% being significantly higher than other classifiers.

Amin et al. [110] combined the structural and texture information of four brain tumor MRI images, T1C, T1, FLAIR, and T2, using a discrete wavelet transform (DWT) and Daubechies wavelet kernel, and then utilized a partial differential diffusion filter to eliminate the redundant noise in the pictures. The global threshold method for segmenting and extracting tumor target regions was used to transport the CNN model to distinguish tumor from non-tumor. The experimental data came from five publicly available datasets, and compared to a single sequence on the benchmark dataset, the experimental results of the fused images provided an accuracy of up to 99% compared to the methods proposed by the BRATS 2013 challenge and the BRATS 2018 challenge.

The CNN developed by Abiwinanda et al. [111] was used to distinguish brain tumor MRI images to reduce human error. Glioma, meningioma, and pituitary tumor are the three most frequent brain tumors identified by the network. The experiment employed 3064 T-1 weighted CE-MRI images, with a training accuracy of 98.51% and a verification accuracy of 84.19%.

Havaei et al. [112] suggested a CNN approach for segmenting MRI images of ischemic stroke lesions and high- and low-grade gliomas automatically. Compared with the existing methods, the convolution characteristics of their model could reach the segmentation of the complete brain image within 3 min, which had a high computational efficiency. Their CNN was trained on image modes to learn the features of representations directly from the data. They also proposed two architectures, one focusing on the small details of glioma, and the other on the larger context.

Zhao et al. [113] developed a new brain tumor segmentation approach based on conditional random fields (CRFs) and complete CNN integration. It came in second place in the challenge dataset and first position in the leaderboard dataset in the BRATS 2013 evaluation. The approach is competitive in decreasing storage costs and data collection when compared to other highly-ranked methods.

Hossain et al. [114] proposed utilizing the fuzzy C-means clustering (FCC) algorithm, a conventional classifier, and CNN to remove brain cancers from two-dimensional MRI of brain images. The experimental dataset was a real-time dataset with different tumor sizes, shapes, locations, and image intensities. They applied the traditional support vector machine methods, multilayer perceptron, naive Bayes, logistic regression, *k*-nearest neighbor, and random forest. In contrast, CNN brought 97.87% accuracy, far exceeding the performance of traditional classifiers.

Pereira et al. [115] presented a CNN for automated segmentation of brain tumors based on MRI images of the brain. They considered a lower number of network weights and used small kernels to achieve deeper network architecture while avoiding overfitting problems. Intensity normalization was employed as a preprocessing step to show the efficiency of data augmentation for MRI brain images on tumor segmentation. In the 2013 Brain Tumor Segmentation Challenge, they won a number of first-place prizes.

Pathak et al. [116] also used small kernels to design a deep network, and used CNN and the watershed algorithm (WA) to find, segment, and classify features in brain tumor images. CNN was tasked with determining whether the photos included cancer. WA segmentation and morphological operation were used to classify pathogenic characteristics of pictures in the presence of malignancies. Experiments suggested that combining CNN and WA could reach an accuracy of up to 98%.

Khan et al. [117] proposed a CNN combining data enhancement and image processing to classify cancerous and non-cancerous brain MRI images. The transfer learning (TL) method was used to compare the performance of their scratch CNN model and the VGG-16, Inception-V3, and Resnet-50 models. In an experiment with small data, the scratch CNN model achieved 100% accuracy compared to the 75% accuracy of Inception-V3, 96% accuracy of VGG-16, and 89% accuracy of RESNET-50. The model had a low complexity rate, less computing power, and higher accuracy.

Deng et al. [118] developed a novel segmentation method for brain tumors by integrating dense microblock differential features (DMDF) and full CNN. The texture feature analysis and local feature extraction of brain images were completed by Fisher vector coding. Finally, the full CNN was added for boundary segmentation. Compared to the old MRI brain tumor segmentation approach, the brain tumor picture can now be segmented in under a second. The average dice index of this model reached 90.98%, which had better segmentation efficiency, accuracy, and stability.

In summary, in the field of brain tumor detection, authors who proposed CNN method, their models and experimental results are shown in Table 5.

### 5.3. Other Regions

Osteoma is also one of the common benign tumors, which is rarely detected in its early stages by patients since it usually occurs in diverse places of the bone, the growth is relatively small, and it is difficult to feel. Sometimes osteomas can lead to headache symptoms, but the symptoms are not too noticeable. When the osteomas develop into large ones, they can quickly press on the nerves in the head and cause deformities in the head, so it is difficult for patients to get treatment.

Barzekar and Yu [119] proposed a CNN architecture, C-NET, which consists of a series of multiple networks to classify biomedical histopathological images from public data sets, including Barzekar and osteosarcoma. Compared with the traditional deep learning model, which uses transfer learning to solve the problem, this model contains multiple CNNs, and the first two parts of the architecture contain six networks of feature extractors to classify tumor images according to malignancy and benignancy. C-NET applied several evaluation indexes to test the reliability of the model and realized zero error classification.

Mishra et al. [120] suggested a CNN that could enhance the efficiency and accuracy of dividing osteosarcoma tumors into tumor and non-tumor categories. The architecture consists of three sets of stacked twoconvolution layers for the maximum pooling layer for feature extraction and two fully linked layers with a data enhancement strategy. This model’s accuracy was 95% higher than the three existing picture categorization algorithms, AlexNet, LeNet, and VGGNet, which proved that CNN could ensure the improvement of high accuracy and high efficiency of osteosarcoma classification.

The number one risk for women is breast tumors. There are many factors contributing to breast tumors, with a high-fat diet and genetic factors being possible inducers, and irregular work and rest and other issues also increasing the risk of breast tumor development in women. Breast tumors occur when many benign masses are developed in the breast, which may be fibromas or cystic growths. But no matter which kind, there is a great possibility of them becoming malignant tumors. As time continues to develop, these tumors will also increase the possibility of cancer. It is best to treat in time.

For the segmentation of breast cancers within the region of interest on mammography, Singh et al. [121] proposed a conditional GAN. Adversarial network learning distinguished real segmentation from synthetic segmentation, and generative network learning would identify tumor regions and create contours. The generative network could create as realistic a situation as possible so that the model would work well even with a small number of training samples. Furthermore, a CNN-based shape descriptor with an overall accuracy of 80% could classify the generated information into four tumor shapes: irregular, lobular, elliptical, and circular.

Ting et al. [122] proposed an algorithm called CNN Improvement for Breast Cancer Classification, with experimental data sets derived from the portable grayscale image format of the Association for Breast Image Analysis. At the same time, the current research results were compared with Neuroph’s commercial software. The accuracy rate was 90.50%. This method effectively ensures the lowest false positive rate, is superior to other methods, and has an outstanding quality parameter.

Bakkouri and Afdel [123] proposed a discriminant target for supervised feature learning to classify tumors on mammograms as malignant or benign by training CNNs. The selected data set input image was set to a fixed length, and the region of interest with a normalized size was obtained based on the Gaussian pyramid. By using geometric transformation technology, the data set could be expanded on the original basis to prevent overfitting. The classification part of the trained CNN model was carried out using the softmax layer. An accuracy of 97.28% was obtained in the experiments on the common data sets DDSM and BCDR. This can assist radiologists in making diagnostic decisions without increasing false negatives.

Wang et al. [124] developed an automated breast volume scanner (ABVS) slice-based computer-aided identification approach for breast cancers. Tumor candidate acquisition, false positive reduction, and tumor segmentation were all included in the system. Breast tumor candidates were first identified using the local phase, and then CNN reduced the number of false positives to improve accuracy and efficiency and avoid complex feature extraction. The experimental data set was derived from clinical ABVS, with radiologists manually marking cases. Delineated breast tumors are segmented by the local binary pattern of superpixels. Experiments proved that using the CNN in false positive reduction stage, 78.12% and 100% sensitivity were obtained according to different FPs/case 0 and 2.16, respectively.

BreastNet was a CNN proposed by Toğaçar et al. [125] for classifying breast cancers using deep learning models. The model was a residual architecture based on the attention module. Enhancement techniques were applied to each image data, which could then be used as input to the model. The attention module selected and processed key areas of the image entering the model. In BreakHis picture data set testing, the model achieved 98.80% classification accuracy, which was higher than that of the AlexNet, VGG-16, and VGG-19 models on the same data set.

Zhang et al. [126] developed an advanced neural network method integrating graph convolutional network (GCN) and CNN to detect malignant lesions in mammography. The suggested algorithm was tested 10 times on a breast mini-MIAS dataset containing 322 mammograms and found to be 96.10 ± 1.60% accurate, with 96.20 ± 2.90% sensitivity and 96.00 ± 2.31% specificity. Compared to 15 state-of-the-art breast cancer detection technologies, it demonstrated superior performance and proven to be successful in the detection of malignant breast masses.

For ultrasound (US) images of breast cancers, Zeimarani et al. [127] suggested a new CNN classification approach. With a total of 641 pictures, the experimental data contained 413 benign lesions and 228 malignant lesions. The tumor classification accuracy was 86.12% after five-fold cross-validation of the model’s classification performance, and the area under the ROC curve (AUC) was 0.934. The implementation of picture enhancement and regularization improved the accuracy rate (92.01%) and AUC (0.9716%), which outperformed other machine learning methods based on human feature selection.

Based on the Inception recurrent residual CNN (IRCNN) model, Alom et al. [128] suggested a method to classify breast cancer. IRCNN combining Inception, recurrent CNN, and residual network is a powerful DCNN model. In the comparison of target recognition tasks, IRCNN showed better performance than equivalent monomial networks. Data from BreakHis and Breast Cancer Classification Challenge 2015 were used. Compared with the existing advanced methods, IRCNN model had excellent scores in AUC, ROC curve, and accuracy, proving that it had a better classification performance.

About half of all malignant tumors are found in the digestive tract. Gastric cancer, esophageal cancer, colorectal cancer, and other malignancies of the digestive tract are common. Long-term drinking, and being fond of spicy and pickled food is the main cause of esophageal cancer. Colorectal cancer is usually associated with a diet high in fat, meat, fiber, and vitamins. Early signs of gastrointestinal cancer include loss of appetite and indigestion. Patients often have abdominal distension, abdominal discomfort, and other problems. Early detection of digestive tract tumors is essential to prevent the development of early gastrointestinal malignancies to late stage.

Hirasawa et al. [129] developed a CNN model based on endoscopic images to classify stomach cancers automatically. Its diagnostic technology was made up of a Single Shot Multibox Detector, which contained 13,584 endoscopic images of stomach cancer that were utilized to train a CNN. The CNN was tested using an independent test set of 2296 stomach photos taken from 77 individuals with gastric cancer lesions, with a sensitivity of 92.2%. Despite the fact that skilled endoscopes were not able to achieve a flawless diagnosis, 161 non-cancerous lesions were nonetheless misdiagnosed.

Li et al. [5] used 386 photos of non-cancerous lesions and 1702 images of early gastric cancer to train an Inception v3 CNN model for analyzing gastric mucosal lesions seen through a narrow-band imaging amplified endoscope. In a performance experiment testing the diagnosis of early gastric cancer, the suggested system’s accuracy was 90.91%, and there was essentially no difference in the system’s specificity and accuracy when compared to the diagnostic ability of endoscopes. CNN systems, on the other hand, have a much higher diagnostic sensitivity than experts.

In summary, the models and applications proposed by the authors for tumor detection of bone tumors, breast tumors and digestive tract tumors by convolutional neural network are shown in Table 6.

## 6. Conclusions

This paper reviews the application of CNNs in tumor detection. The content includes the development history of computer-aided diagnosis of tumors, the basic working principle and structure of tumor image classification diagnosis based on CNN, the introduction of classical structures, and the application in medical image analysis of different types of tumors. Firstly, the basic methods of computer-aided tumor diagnosis are summarized, including multiple image feature extraction methods, feature reduction, and categorization. The CNN is proposed to improve the quality of computer-aided tumor diagnosis. The fundamental layer architecture, activation function, loss function, gradient descent, feedforward, feedback propagation, and the basic layer architecture, activation function, and loss function are all discussed. The primary benefits and drawbacks of numerous traditional architectures are introduced, including AlexNet, VGGNet, GoogleNet, and ResNet. Finally, we summarize some of the applications of CNNs in lung tumors, brain tumors, and other types of tumors, including osteosarcoma, breast tumors, and gastrointestinal tumors.

Common medical images of tumors provide a great deal of useful information for medical images of cancer. In terms of accurate and effective use of image information, the CNN-based computer-aided diagnosis method is superior to the traditional learning effect in tumor segmentation and classification. It can extract more significant features from data sets automatically, and the information extraction procedure is simplified. It can assist doctors in supplementing clinical decision-making by converting qualitative, subjective picture information into quantitative objective image information.

## Figures and Tables

**Table 1:** A list of imaging examination methods commonly used in clinical oncology.

Methods	Advantages	Drawbacks	Application
X-ray radiography	X-ray imaging has apparent advantages in examining dynamic and subtle lesions, especially in bone, gastrointestinal, vascular, breast, and other examinations.	X-rays are harmful to the human body due to the radiation. The contrast resolution of film X-ray images is low, and the identification ability of soft tissue is poor.	Medical X-ray diagnostic equipment is the earliest and most widely used medical imaging examination means.
Computed tomography	CT images can show a cross-section of a part of the body, and the shape and density of organ tissues are displayed. Its density resolution is better than an X-ray image.	In examining brain tissue near the bone wall, CT imaging is not as good as MRI due to the interference of bone.	CT can be used to diagnose cancer with intracranial tumors or cardiovascular lesions without MRI.
Magnetic Resonance Imaging	Magnetic resonance imaging is fast and carries a low risk of injury. The imaging effect of the nervous system, cartilage, and muscle tissue in the body is excellent.	The price of MRI examination is relatively high, with a long examination time, and patients feel bad during the process of MRI examination.	MRI can clearly diagnose brain tumors, bone tumors, and so on, especially for brain tumor diagnosis, and is obviously superior to CT.
Ultrasound Color Doppler	It is low cost, convenient, and affordable, with no radiation or other adverse effects. It has an obvious accuracy advantage in the examination of dynamic and subtle lesions.	Usually, the image quality is poor, and it is difficult to obtain accurate boundaries of cancer areas and identify small nodules.	UCD detects the texture and density of body tissues. It is used for the examination and diagnosis of heart, limb blood vessels, and superficial organs, as well as abdomen and obstetrics and gynecology.
Computer-aided diagnosis system	The computer objective classification corrects the diagnostic problems that may be caused by the limitation and influence of the knowledge level of doctors in the subjective identification process.	Human intervention is unavoidable. Most diagnostic systems are limited and can only detect a single model.	It is mainly used for breast and chest pulmonary nodular diseases, but seldom for CT diagnosis of liver disease or MRI diagnosis of brain tumor.

**Table 2:** All layer parameters for AlexNet.

Layer-Name	Kernel-Size	Kernel-Num	Padding	Stride
Conv1	11	96	[1, 2]	4
Maxpool1	3	None	0	2
Conv2	5	256	[2, 2]	1
Maxpool2	3	None	0	2
Conv3	3	384	[1, 1]	1
Conv4	3	384	[1, 1]	1
Conv5	3	256	[1, 1]	1
Maxpool3	3	None	0	2
FC1	2048	None	None	None
FC2	2048	None	None	None
FC3	1000	None	None	None

**Table 3:** The network structure of GoogLeNet.

Type	Depth	Stride	Size(output)	#1 × 1	#3 × 3 Reduce	#3 × 3	#5 × 5 Reduce	#5 × 5	PP	Params
Conv	1	7 × 7/2	112 × 112 × 64							2.7 K
Max pool	0	3 × 3/2	56 × 56 × 64							
Conv	2	3 × 3/1	56 × 56 × 192		64	192				112 K
Max pool	0	3 × 3/2	56 × 56 × 192							
Inception (3a)	2		28 × 28 × 256	64	96	128	16	32	32	159 K
Inception (3b)	2		28 × 28 × 480	128	128	192	32	96	64	380 K
Max pool	0	3 × 3/2	14 × 14 × 480							
Inception (4a)	2		14 × 14 × 512	192	96	208	16	48	64	364 K
Inception (4b)	2		14 × 14 × 512	160	112	224	24	64	64	437 K
Inception (4c)	2		14 × 14 × 512	128	128	256	24	64	64	463 K
Inception (4d)	2		14 × 14 × 528	112	144	288	32	64	64	580 K
Inception (4e)	2		14 × 14 × 832	256	160	320	32	128	128	840 K
Max pool	0	3 × 3/2	7 × 7 × 832							
Inception (5a)	2		7 × 7 × 832	256	160	320	32	128	128	1072 K
Inception (5b)	2		7 × 7 × 1024	384	192	384	48	128	128	1388 K
Avg pool	0	7 × 7/1	1×1 × 1024							
Dropout (40%)	0		1 × 1 × 1024							
Linear	1		1 × 1 × 1000							1000 K
Softmax	0		1 × 1 × 1000							

**Table 4:** Results of the application of convolutional neural networks in the lung tumor area.

Authors	Model	Results				
		Accuracy	Sensitivity	Specificity	Mean Dice	Median Dice
Zhu et al. [88]	Deeplung	81.41%	-	-	-	-
Vijh et al. [89]	WOA_APSO	97.18%	97%	98.66%	-	-
Lu [90]	DCNN	96.4%	97.6%	95.2%	-	-
Rani and Jawhar [91]	BDCNN	97.3%	94.9%	100%	-	-
Teramoto et al. [92]	DCNN	87.0%	89.3%	83.3%	-	-
Shi et al. [93] (iteration 10 times)	CNN	94.28%	-	-	-	-
	PET-CNN	97.43%	-	-	-	-
	PET/CT-CNN	95.45%	-	-	-	-
	Integrated CNN	99.44%	-	-	-	-
Hossain et al. [94]	U-Net	-	-	-	58.48%	62.29%
	LungNet	-	-	-	62.67%	66.78%
	Dilated CNN	-	-	-	65.77%	70.39%
Wang et al. [95] (iteration 10 times)	CT-CNN	96.67%	96%	97.33%	-	-
	PET-CNN	98.67%	99.33%	97%	-	-
	PET/CT-CNN	97%	95.33%	98.67%	-	-
	Ensemble CNN	99.33%	99.33%	99.33%	-	-
Tahmasebi et al. [96]	Fully CDNN	-	-	-	91%	-
Lin et al. [97]	GAN-AlexNet	99.9%	99.9%	100%	-	-
Nair et al. [98]	Fully DCNN	90.32%	92.3%	80%	91%	-
Teramoto et al. [99]	FP-reduction CNN	-	90.1%	-	-	-
Gan et al. [100]	Hybrid CNN	-	-	-	72%	-
Nishio et al. [101]	Conventional method	55.9%	-	-	-	-
	DCNN	62.4%	-	-	-	-
	DCNN (TL)	68%	-	-	-	-
Moitra and Mandal [102]	CNN-RNN	97%	-	-	-	-

(Acc = accuracy; Sen = sensitivity; Spc = specificity).

**Table 5:** Results of the application of convolutional neural networks in the brain tumor area.

Authors	Model	Results			
		Accuracy	Dice		
			Complete	Core	Enhanced
Toğaçar et al. [106]	BrainMRNet	96.05%	-	-	-
Sajjad et al. [107]	DCNN	90.67%	-	-	-
Saxena et al. [108]	CNN-TL	95%	-	-	-
Pashaei et al. [109]	KE-CNN	93.68%	-	-	-
Amin et al. [110]	DWT-CNN	99%	-	-	-
Abiwinanda et al. [111]	CNN	84.19%	-	-	-
Havaei et al. [112]	CNN	-	76%		
Zhao et al. [113]	FCNN-CRF	-	86%	73%	62%
Hossain et al. [114]	FCC-CNN	97.87%	78%	65%	75%
Pereira et al. [115]	CNN	-	88%	83%	77%
Pathak et al. [116]	CNN-WA	100%	-	-	-
Khan et al. [117]	CNN-TL	100%	-	-	-
Deng et al. [118]	FCNN-DMDF	-	91%		

**Table 6:** Results of the application of convolutional neural networks in other tumor fields.

Pathological Type	Authors	Model	Results	
			Accuracy	Sensitivity
Osteoma	Barzekar and Yu [119]	C-Net	99.34%	-
	Mishra et al. [120]	CNN	92.4%	-
Breast tumors	Singh et al. [121]	cGAN-CNN	80%	-
	Ting et al. [122]	CNNI-BCC	90.5%	-
	Bakkouri and Afdel [123]	CNN-softmax	97.28%	-
	Wang et al. [124]	ABVS-CADe	-	100%
	Toğaçar et al. [125]	BreastNet	98.8%	-
	Zhang et al. [126]	BDR-CNN-GCN	96.1%	96.2%
	Zeimarani et al. [127]	CNN-US	92.01%	-
	Alom et al. [128]	IRRCNN (binary)	99.05%	-
		IRRCNN (multi-class)	98.59%	-
Digestive tract tumors	Hirasawa et al. [129]	SSD	-	92.2%
	Li et al. [5]	CNN-M-NBI	90.91%	-

## Data Availability

Not applicable.
